# Development and Application of MiMouse, a Comprehensive Genomic Profiling Panel for Credentialing Mouse Tumor Models

**DOI:** 10.1158/2767-9764.CRC-25-0279

**Published:** 2025-10-29

**Authors:** Kevin Hu, Chia-Jen Liu, Zhaoping Qin, Aaron M. Udager, Marcin P. Cieslik, Scott A. Tomlins

**Affiliations:** 1 Department of Computational Medicine & Bioinformatics, University of Michigan Medical School, Ann Arbor, Michigan.; 2 Department of Pathology, University of Michigan, Ann Arbor, Michigan.; 3 Department of Internal Medicine, University of Michigan Medical School, Ann Arbor, Michigan.; 4 University of Michigan Rogel Cancer Center, Ann Arbor, Michigan.; 5 Michigan Center for Translational Pathology, University of Michigan, Ann Arbor, Michigan.; 6 Department of Urology, University of Michigan Medical School, Ann Arbor, Michigan.

## Abstract

Despite shared genetic driver alterations and histology, the genomic fidelity of most mouse tumor models, including those genetically engineered (GEMM), to their human counterparts is unknown. In this study, we developed MiMouse, a mouse comprehensive genomic profiling panel for high-throughput credentialing applicable to routine formalin-fixed, paraffin-embedded tumors. Through simulation/validation, we focused on considerations for cross-species mutation prioritization, strain determination, and aneuploidy detection. Using MiMouse, we profiled >250 tumors from high-grade serous carcinoma GEMMs based on conditional inactivation of *Brca1* (*B*), *Trp53* (*P*), *Pten* (*Pt*), *Rb1* (*R*), and/or *Nf1* (*N*) and a colorectal carcinoma GEMM based on conditional inactivation of *Apc*, *Kras*, and/or *P*. We confirmed increased genomic instability in high-grade serous carcinoma tumors, with *BPPt* cancers having both the shortest latency and the least genomic instability. In colorectal cancer, focusing on fidelity to human colorectal cancer aneuploidy events, our results highlighted the critical importance of synteny in transgenic studies, as not only was loss of mouse chromosome 18 (containing the tumor suppressor gene *Smad4*) a significant aneuploidy event (18%), additional tumors harbored focal *Smad4* copy loss, potentially due to the mouse-specific proximity of *Apc* (mouse and human chromosomes 18 and 5, respectively). Likewise, mouse chromosome 5, the only significantly gained (46%) chromosome in our colorectal cancer models, has syntenic blocks from human chromosomes 7p, 7q, and 13q, including *Cdx2*, which is both a lineage-specific colorectal cancer oncogene and the colorectal cancer GEMM promoter source. Given the importance of mice to translational cancer research, this study highlights the considerations and utility of approaches for comprehensive genomic credentialing.

**Significance:**

The genomic fidelity of most mouse tumor models is unknown. Considering cross-species issues, we develop MiMouse for high-throughput genomic credentialing and profile >250 tumors from fallopian tube and colorectal tumor models.

## Introduction

Mice are the primary model organism used in cancer research due to their quick reproductive capabilities, molecular similarities to humans, and capacity for transgenic model creation (1–3). Murine models vary by how neoplasia is introduced (e.g., exposure to mutagens and introduction of oncogenic transgenes), with differing ease of generation, utility, genomic fidelity, and limitations. For instance, in genetically engineered mouse models (GEMM), tumorigenesis is induced using genetic methods (e.g., CRISPR or Cre-Lox) to activate oncogenes or inactivate tumor suppressor genes (TSG) in all cells or targeted cell populations using tissue-specific promoters. GEMMs provide the means with which to study *de novo* tumorigenesis and neoplastic progression in the context of an intact and co-evolving immune system (4). Common limitations of GEMMs are the lengthy production times, influence of background strain composition, and forced introduction of a limited set of driver alterations that do not reflect the full spectrum of those relevant to each tumor type. Furthermore, these genetic drivers may not genomically model the timing, sequence, or background exposures that likely play important roles in human tumorigenesis (5–7).

Although the advent of next-generation sequencing (NGS) has enabled large whole-exome and/or whole-genome sequencing (WES/WGS) studies resulting in a near saturation in discovery of recurrent germline and somatic mutations, copy-number alterations (CNA), and gene fusions for most human tumors (8), the vast majority of these alterations have been known for many decades and have been used in the creation of tumor type–appropriate GEMMs driven by a limited number of such alterations (1–3). More recent studies, particularly those using WGS and integrating findings across tumor types, have also led to the characterization of genome-wide mutations, CNA, and structural variant signature, which have demonstrated tumor type–specific co-occurrence patterns of these alteration classes (9–17). Importantly, although examples of true “pan-tumor” functional effects of specific genomic alterations exist (e.g., nearly all tumors driven by *NTRK1/2/3* fusions are sensitive to small molecular NTRK inhibitors), the vast majority of oncologic alterations have profound tumor type dependencies in either their occurrence or impact (e.g., melanomas with *BRAF* p.V600E mutations are highly sensitive to single-agent BRAF/MEK inhibition, whereas colorectal cancers with the same *BRAF* p.V600E are not; ref. 18), suggesting that the most relevant GEMMs must have high fidelity to the corresponding human tumor cell of origin and/or genomic context.

Critically, although many GEMMs definitionally show high fidelity to recurrent single-gene oncogenic and tumor suppressor alterations, whether most models show high genomic fidelity to their human tumor counterparts in some, or all, relevant genomic alteration classes is largely unknown. Importantly, with mice being approximately 3,000× smaller than humans, mouse tumor precursors and even established tumors often yield only minute amounts of DNA (like human tumors submitted for clinical testing), which can be problematic for routine WES/WGS approaches (19–21). Likewise, precise microdissection guided by pathologists on formalin-fixed, paraffin-embedded (FFPE) tumor specimens is frequently required in human tumor NGS to ensure appropriate tumor content (22, 23); this issue is even more relevant when attempting to profile the spectrum of neoplasia from initial precursor lesions to distant metastases (24). Additionally, inter-observer disagreement on tumor content by routine histopathology is frequent (25), with tumor content having a dramatic impact on the depth (and cost) of sequencing required for both basic and comprehensive genomic characterization.

Given these limitations, most GEMMs have not been comprehensively assessed for genomic fidelity to human tumors. For instance, aneuploidy [gains or losses of entire chromosomes (chr) or chr arms] is one of the most common alterations found in human tumors (occurring in more than 88% of human solid tumors) with distinct patterns occurring across tumor types (11, 16, 17, 26). Recently, through systematic assessment of aneuploidy in human tumors coupled with functional studies, Girish and colleagues (27) showed that *TP53* mutations and chr 1q gain occurred less frequently than expected by chance, with functional studies demonstrating that although *MDM4* (1q32.1) was the major functional driver of the 1q aneuploidy event, *BCL9* (1q21.2) was also a dosage-sensitive driver, supporting the importance of multiple genes in at least some recurrent aneuploidy events. Notably, in GEMMs that have been characterized, distinct patterns and degrees of aneuploidy have been observed; however, whether these aneuploidies affect the same genic regions in human tumors has been under-explored (7, 26, 28). Importantly, because the organizational structure of the human and mouse genome vastly differs (29), the use of syntenic blocks (mapping of blocks of ordered orthologous genes between two species) may help elucidate which genes/regions of aneuploidy events drive neoplastic progression (26, 28). For example, *Mdm4* and *Bcl9* map to different mouse chrs (chr 1 and 3, respectively), suggesting that high-fidelity mouse models of this aneuploidy event might be expected to show gain of both genes and/or chrs, which could then be functionally evaluated through separate manipulation of observed events.

GEMMs also have unique genomic fidelity aspects that must be considered, such as strain composition. Historically, GEMMs with single-gene knockouts were created using the embryonic stem cell approach in the 129S1/SvmJ strain and were then backcrossed to the desired strain (6, 30). More complex models like multi-gene knockouts can lead to a GEMM consisting of multiple strains, which are not backcrossed to maintain their desired genotype (31). The knowledge of strain admixture is critical, as it can be a confounding factor when analyzing phenotypic differences. Taken together, these observations demonstrate the need for comprehensive genomic characterization of tumors arising in different GEMMs to evaluate their fidelity to human tumorigenesis, as highlighted by the challenges associated with the assessment of minute lesions from FFPE tissue. WES/WGS is infrequently performed clinically in human tumors for the reasons described above; however, clinical testing has progressed from small panels (10–50 genes) targeting primarily oncogenes to larger panels targeting hundreds of oncogenes/TSGs across hundreds of kilobases (kb)/megabases (Mb). This comprehensive genomic profiling (CGP) enables assessment of nearly all known driver genes, genomic variant classes, and their co-occurrence, with the minimal clinical tradeoff of less genomic detail than WES/WGS (32–34). Importantly, we and others have shown that amplicon-based approaches enable CGP with more limited input DNA obtained after precise microdissection of human precursor lesions from FFPE tissue (23, 35–39).

Human colorectal carcinoma and “ovarian” high-grade serous carcinoma (HGSC) highlight the importance of genomic fidelity in GEMMs. The histologic and molecular neoplastic progression of human colorectal carcinoma has been well defined for over 30 years (40), typically beginning with biallelic *APC* inactivation (to form precursor adenomas), followed by sequential acquisition of activating *KRAS* mutations and *TP53* inactivating alterations, and chr 18q loss during the transition to overt adenocarcinoma. Numerous colorectal carcinoma GEMMs have been developed (41–43), from random mutagenesis-induced *Apc* inactivation in the *Apc*^Min^ model characterized more than 30 years ago, to those precisely mirroring key human alterations (e.g., colon-specific conditional expression of biallelic *Apc* inactivation and *Kras* p.G12D mutation, with combinations of *Trp53* mutation/loss and/or *Smad4* inactivation; refs. 44, 45). Notably, however, colorectal carcinoma also shows highly recurrent, aneuploidy gains of chr 20q, 13q, 7q, and 8q (46–48), which have not been extensively evaluated in the context of such GEMMs. Likewise, although the initiating alteration (*TP53* mutation) in HGSC has been known for decades, early attempts at developing mouse models targeted this alteration to ovarian surface epithelium (OSE; refs. 49, 50). However, the recognition that HGSC typically arises from the fallopian tube epithelium (FTE) and is genomically characterized by (i) a striking lack of other recurrent somatic mutations, focal high-level CNAs, or gene fusions and (ii) profound genomic instability (51–55) required the development, characterization, and verification of genomic fidelity of novel HGSC GEMMs based on neoplastic transformation of oviductal epithelium (vs. OSE).

Our interest in this area began with an effort to support the initial genomic assessment of a histologic high-fidelity mouse model of HGSC based on conditional inactivation of TSGs frequently inactivated in human HGSCs, including *Brca1* (B), *Trp53* (P), *Nf1* (N), *Rb1* (R), and *Pten* (*Pt*), in the FTE. Using tamoxifen-regulated Cre recombinase under control of the *Ovgp1* promoter (*Ovgp1-iCreER*^*T2*^; ref. 56) to inactivate *Brca1*, *Trp53*, *Rb1*, and *Nf1* (hereafter referred to as *BPRN*) or *Brca1*, *Trp53*, and *Pten* (hereafter referred to as *BPPt*), we generated GEMMs that reliably acquire oviductal HGSCs. An HGSC-focused, FFPE-compatible, targeted mouse NGS panel (156 kb; ref. 31) was developed to evaluate driver mutations and focal high-level CNAs in the mouse orthologs of the 32 most significantly altered genes in human HGSC as identified in The Cancer Genome Atlas (TCGA) assessment of ovarian cancer (52, 57). Although this panel was able to verify recombination of the targeted TSGs and identify rare somatic gene alterations (e.g., biallelic *Pten* deletions and *Myc* amplifications) in FFPE mouse tumor samples, it lacked the capability to comprehensively evaluate genome-wide fidelity for more complex alteration classes (31). Hence, herein, to enable high-fidelity genomic credentialing of FFPE GEMM tumors across all stages of neoplasia and different tumor types, we considered the requirements for such a mouse CGP panel and applied our resulting mouse CGP panel (MiMouse) to a large cohort of HGSC and colorectal carcinoma GEMM tumors (spanning precursor lesions through metastases). In addition to highlighting general considerations for mouse CGP development, our results further credential these HGSC and colorectal carcinoma GEMMs and particularly highlight the importance and utility of synteny-based cross-species analyses to prioritize driver genes/regions from recurrent aneuploidy events.

## Materials and Methods

### MiMouse panel design: overview

A custom-targeted panel for murine FFPE-compatible CGP was created through the Life Technologies AmpliSeq service. Balancing throughput and panel size, a total of 135 genes [targeting hotspots, CNAs, and/or the full coding sequence (CDS)] and tumor suppressors (full CDS) were selected by manual review of human CGP panels and our previous HGSC-focused targeted murine panel (31). For genes assessed only for CNAs, a minimum of 10 amplicons (when possible) were included (38). Amplicons chosen for tiling CNAs were chosen to be equally dispersed between exons using a custom R script. For hotspot mutations of interest, a custom script was used to convert genomic locations between hg38 and mm10 genome assemblies. Another custom script utilizing a local conservation score was used to convert between the two species (see below). After selection of additional 500 non-genic SNP targets for genotyping, human genic targets were converted to mouse genomic coordinates using both UCSC’s LiftOver (RRID: SCR_018160) and the R package biomaRt (RRID: SCR_019214; ref. 58) as described below. These amplicons together with the converted hotspot mutations and genotyping SNPs were made into a bed file and submitted to Life Technologies Ion Torrent custom panel creator (the TSGs can also be entered separately into the website as Ion Torrent has precalculated amplicons to tile the genes’ full CDS; ref. 59). The resulting final designed MiMouse panel included 4,262 amplicons in two pools targeting 502 kb.

### MiMouse panel design and simulation: hotspot mutation conversion

Mutation data from Zehir and colleagues (60) were acquired through cBioPortal and annotated using oncokb-mafannotator. A custom script was used in the conversion of hotspot mutations. Briefly, LiftOver and chain files obtained from the UCSC genome browser (RRID: SCR_005780) were used to convert all human coordinates to hg38 before mapping to mm10 coordinates (29, 61). BiomaRt was used to create custom tables to query Ensembl annotations, CDS mapping information, genomic coordinates, APRISS annotations, and peptide sequences. Using the custom tables, each amino acid (aa) position was converted into its genomic coordinates (hg38) using CDS information and mapped to mm10 using LiftOver. The reverse process was done to convert the mm10 coordinates into the aa positions. Residues of interest were obtained by using the peptide sequences associated with the respective Ensembl transcript. Based on the CDS, every human and mouse Ensembl transcript was considered when looking for conserved mutations in each gene. Sequence alignment was done using the R package msa and with the VTML10 substitution matrix (62). VTML10 was chosen as we assumed high percent identity (∼90%) in genes frequently mutated in cancer. The alignment was performed on the aa position of interest and 14 aas surrounding it, which was based on alignment lengths calculated for the VTML10 scoring matrix (63). The workflow was tested on all 4,155 prioritized aa positions (aas) between the 270 available genes designated as either tumor suppressors or oncogenes found in OncoKB. Shared hotspots had to meet two criteria: (i) the aa of the specified position is matched between the two species and (ii) the mean local alignment score was greater than 0 (used to filter out spurious matching aa occurring by chance). Human transcripts with a MANE-SELECT annotation were subsequently used for downstream analysis as MANE-SELECT transcripts are the standard transcript used by Catalogue of Somatic Mutations in Cancer (RRID: SCR_002260). Two factors were used when considering which mouse Ensembl transcript (per gene) to use: the number of conserved positions mapped to the human MANE-SELECT transcript and APRISS annotation. For aa positions not conserved between the MANE-SELECT transcript and designated mouse transcript, an alternative transcript in which the position was found to be conserved was used. Multiple regression, standardized ORs, and forest plots were analyzed in R using the packages *glm*, *effectsizes*, and *forest_model* (64, 65). Effect sizes were made comparable between continuous and categorical variables by scaling variables by two SDs and centering to the median. *Trp53* was excluded from the *glm* analysis as its mutational features differ from all other TSGs (it has an abundance of hotspot mutations vs. usual deleterious mutations; ref. 66). Custom Pfam annotations from Interpro version 5.73 were used to map the aa positions to their respective protein domains (67, 68).

### MiMouse panel design and simulation: genotyping SNPs

The set of 1,638 SNPs identified by Petkov and colleagues (69) as informative in building phylograms on 102 tested mouse strains was used to select those for inclusion in MiMouse. The 1,638 SNP set was first reduced by filtering out non-informative SNPs (zero entropy) from the five following strains we aimed to assess by genotyping: C57BL/6J (RRID: IMSR_JAX:000664), BALB/cJ (RRID: IMSR_JAX:000651), C3H/HeJ (RRID: IMSR_JAX:000659), FVB/NJ (RRID: IMSR_JAX:001800), and 129S1/SvmJ (RRID: IMSR_JAX:002448). Mm10 coordinates were then obtained by querying the rsIDs in biomaRt (58). Next, as vcf/PLINK files were not available from the Petkov and colleagues study (precluding principal component analysis), we performed multiple correspondence analysis (factor analysis; ref. 70) with a matrix of the strain by variant allele as input. For the four dimensions separating the above five strains, we used the percentage of contribution for each dimension to reduce the set of SNPs, evaluating the predictive function and stability by inferring admixture on simulated mouse samples. These simulations were based on pairwise crossing of each of the five strains creating an F1 generation that was 50/50 admixed. Briefly, bam and vcf files were created for each mouse strain by aligning the respective fastq files from the Mouse Genome Project to the mm10 reference genome and force calling the genotyping SNP positions using bcftools (71). These vcfs were then read into R using vcfR, and in a pairwise fashion between the strains, a random allele was taken for each position to create the admixed mouse (72). Because the original list of 1,638 SNPs had on average 1.5 Mb between each marker, linkage was not expected to influence the simulated genotypes. ADMIXTURE (RRID: SCR_001263) was used on the simulated F1 vcfs (repeated 100 times) to infer the strain estimations (73). Structure plots for the population inferences (averages) were then plotted using ggplot2 (RRID: SCR_014601; ref. 74). For the final MiMouse panel design, we selected a total of 500 non-genic SNPs, balancing panel size versus genotyping resolution (see “Results”). Life Technologies filtered out 10 SNPs because of SNPs being located in regions in which the technology could not accurately measure.

### Simulating aneuploidy and large focal CNAs

Aneuploidy simulations initially tested our metric for coverage-specific CNA detection, *R*_*c*_, and then further tested other factors like marker density. Testing of *R*_*c*_ as a threshold was done using custom R scripts utilizing the R package GenomicRanges to simulate different breadth of coverage (*c*) for chr arms, with each range centered at the midpoint of a chromosome arm, and calculating the overlaps (*R*_*c*_) from TCGA segmentation calls. Chr arm information for hg19 was retrieved from UCSC genome browser. The dataset we used to test *R*_*c*_ as a threshold comprised 6,591 diploid TCGA samples aggregated through cBioPortal, with diploid tumors having a ploidy between 1.6 and 2.3 from ploidy estimations made by ABSOLUTE (RRID: SCR_005198). We tested different *R*_*c*_ thresholds to call aneuploidy ranging from 0.1 to 1.0 with a range of *C* from 0.1 to 1.0. Reference calls were made using the definition of aneuploidy by Taylor and colleagues (17) in terms of our metric *R*_*c*_ with the *R*_*c*_ threshold being an *R*_*1.0*_ ≥ 0.8. Performance metrics [positive percent agreement (PPA)] were calculated using the R package caret (75). Marker density was simulated on nine diploid Cancer Cell Line Encyclopedia (CCLE) colorectal carcinoma samples and five fibroblasts cell lines as normal samples sequenced by the BROAD using WES (76). Samples sequenced with WES were chosen because of the assay’s design being a more comprehensive version of most CGPs: markers were designed to target all genes. From custom R scripts, chr arms from hg19 (UCSC) were split into different non-overlapping windows of sizes 100, 250, 500 kb, 1, 5 and 10 Mb, and random markers in the form of both single marker/whole genes, prioritizing a gene when possible, were randomly sampled per window from the Agilent coverage file. The subsampling was repeated 10 times for each sample and genomic window to account for variability. CNVkit (RRID: SCR_022620) was then used to segment the bam files using the reduced be files using a pool of normals (fibroblast; *n* = 5) and aneuploidy status was called using varying *R*_*c*_ thresholds. A pipeline was created to output copy-number (CN) segment (cns) and CN ratio (cnr) files based on the same filtering and segmentation steps as the CNVkit’s default batch command in order to force the sex of a sample (if known), ploidy, purity, and thresholds (for CN calling). Only the bam files segmented using all WES targets utilized off-target reads (anti-target file), and off-target reads were excluded from the subsampled beds. As above, an *R*_*c*_ threshold of *R*_*1.0*_ ≥ 0.8 was used to call reference set for comparison based on segmentation calls for the full bam files of the CCLE samples.

To improve aneuploidy detection performance with sparser markers, we also filtered aneuploidy calls based on proportion of genes altered in the same direction as an aneuploidy called to control for a high false-positive rate. Instead of using the segment-based gene calls from CNVkit, we called each gene by taking the cnr file and then taking the median of the log_2_CNR of all bins measuring the same gene. Gene calls were then made for each subsampled bam and were used to filter out aneuploidy calls based on the proportion of genes altered in the same direction ranging from 0.3 to 0.5 (directionality filter). The subsequent aneuploidy calls were then used to calculate PPA and true positive rate as described above.

### Mouse tumor samples

*Ovgp1*-*CreER*^*T2*^ (MMRRC_043616-UCD) mice with various tumor suppressor allele modifications and tumors induced in the oviduct by tamoxifen have been described previously (RRID: IMSR_JAX:026563, RRID: IMSR_JAX:008462, RRID: IMSR_JAX:008183, RRID: IMSR_JAX:017835, RRID: IMSR_JAX:017639, RRID: IMSR_JAX:006440, and RRID: IMSR_JAX:003724; ref. 31). Carcinosarcomas (also known as malignant mixed Mullerian tumor) were considered a variant of HGSC and were treated as such in the downstream analysis as we did previously (31). *Cdx2P*-*CreER*^*T2*^ (RRID: IMSR_JAX:022390) mice with *Apc*^*flox/+*^ and *Kras*^*LSL-G12D/+*^ (*AK*) and various *Tp53* (*P*) alterations (*Cdx2P-CreER*^*T2*^*AKP*^*270/+*^, *CDX2P-CreER*^*T2*^*AKP*^*270/fl*^, and *CDX2P-CreER*^*T2*^*AKP*^*fl/fl*^; RRID: IMSR_JAX:009045, RRID: IMSR_JAX:008179, RRID: IMSR_JAX:008462, and RRID: IMSR_JAX:008651) and colorectal tumors and organoid generation have been described in detail previously (45). FFPE, fresh frozen, or paraformaldehyde-fixed tumor samples were obtained at necropsy from grossly involved organs and/or using light microscopy of hematoxylin and eosin–stained sections. All animal husbandry and experimental procedures were carried out under approval from the University of Michigan’s Institutional Animal Care and Use Committee (PRO00007181 and PRO00008343) and according to Michigan state and US federal regulations.

### MiMouse CGP: gene-level and aneuploidy calling

Multiplexed barcoded libraries were made from 20 ng of DNA isolated from FFPE mouse tissue using AmpliSeq Library Kit 2.0 (Life Technologies), sequenced on Ion Torrent Proton or S5XL sequencers, and aligned to mm10, and read counts were quantitated using TMAP (RRID: SCR_000687) in Torrent Suite as described (31). Sample-level quality control (QC) was performed using minimum sequencing uniformity, on target percentage, and mean depth as previously described (31, 38). Somatic variants were detected as described (31).

Log_2_ CN ratio (CNR) for all amplicons was determined and CNAs were detected as described (31), except any samples with (i) an median absolute pairwise difference threshold of >0.50 (to detect unreliable CN profiles) or (ii) less than 30% tumor content by integration of light microscopy tumor content estimation, and log_2_ CNR of floxxed alleles (*Trp53* for HGSC; *Apc* for colorectal carcinoma) was excluded from all CN analyses (39).

Read count data generated from the coverageAnalysis plugin were used to call gene-level somatic CNAs using a custom bed file separating the flox regions from their respective genes (*Apc*, *Trp53*, *Brca1*, *Rb1*, *Nf1*, and *Pten*). Read count data were first filtered by dropping the bottom fifth percentile of amplicons. Read counts were then normalized by total read counts per sample. CNRs were calculated for each amplicon by dividing by the corresponding amplicon in the weighted pooled normal. The pooled normal consisted of tail and liver DNA from mice of both the colorectal carcinoma and HGSC GEMMs. The ratios were then normalized per sample by Loess smoothing of GC content. *Z*-scores were then calculated per gene from the pool of normals and converted into *P* values (two-tailed) using a standard normal distribution. Benjamini–Hochberg correction was then used to correct for FDR. The resulting gene-level calls were then plotted as CN plots (per sample) and heatmaps using custom R scripts with the latter using the pheatmap (RRID: SCR_016418) package. All data were transformed using a logarithm of base 2 (log_2_ CNR) prior to graphing and ordered according to genomic order (77). The heatmap data were additionally filtered for noise by zeroing any gene-level calls with a *q* value > 0.05 and −0.2 < log_2_CNR < 0.2. Clustering of the heatmaps was done on a sample (row) by gene (column) matrix using complete linkage excluding any of the flox alleles. The R package dextend was used to rotate branches from the hierarchical clustering (78).

Segmentation data for aneuploidy calling were performed by using the R package DNAcopy (RRID: SCR_012560) based on the log_2_ CNR data (amplicon) produced in the previous gene-level calls. Data were smoothed and segmented with default parameters (79). The resulting segmentation calls were assessed for significance in a similar fashion as described above for gene-level calls by calculating *Z*-scores based on the amplicons that make up the segment compared with the amplicons from the pool of normal samples. The segments were subsequently filtered based on their *q* value and log_2_ CNR like the gene-calling portion. Because MiMouse’s per arm breadth of coverage ranged from 0.84 to 0.92 we decided to set *C* at 0.6 and used an *R*_*0.6*_ ≥ 0.8 as a *R*_*c*_ threshold to call aneuploidy because different *R*_*c*_ thresholds had the same performance (PPA) after applying our directional filter for genes described below. Briefly, a custom R script was used to create the expected genomic ranges of where the mouse chr were to be detected by downloading the mm10 cytoband files from UCSC and using the R package GenomicRanges (RRID: SCR_000025) to create different ranges of *C* from each mouse chr’s midpoint, with *C* ranging from 0.4 to 0.8 (75). For each chr per sample, we summed up nonzero segments of the same direction and overlapped them with the respective reduced chr GRange described previously (based on *C*). If the overlap was above our *R*_*c*_ threshold, then it was called an aneuploidy. Unlike the simulations using WES, MiMouse has comparably fewer genes, so when using our directionality filter (gene-filter), we required an aneuploidy call to have all genes on the segment altered in the same direction.

Samples sequenced on MiMouse for validation for comparison to Mouse Genotyping Array (MGA) had their log_2_ CNR scaled to for comparison. Scaling was done by taking the ASCAT estimation ploidy and purity and scaling the log_2_ CNR, with and without rounding to the nearest integer (80). All plots made for comparison were done using a custom R script utilizing ggplot2. All statistics were calculated in R (version 4.2.3).

### Identification of somatic variants

Somatic variants were called using Ion Torrent Suite’s variant analysis plugin. Variant call files produced by the plugin were then annotated using AnnoVar (RRID: SCR_012821; ref. 81). Variants were filtered for noncoding variants, indels, and SNPs using the mouse genome project’s dbSNP142 (82). As described, additional empirically determined Ion Torrent–specific filters were used to call variants such as requiring the flow variant allele strand ratio (flow-corrected supporting alternate allele forward reads/flow-corrected supporting alternate allele reverse reads) to be between 0.2 and 5.0, flow-corrected read depth >100, flow-corrected alternative allele reads >10, homopolymer run <4, and an allele fraction >0.1 (31, 38).

### MiMouse analyses: sub-gene comparisons, fraction of genes altered, and copy-level score

Sub-gene analyses pertained to the deletions induced by the Cdx2-P or Cre-mediated systems of our colorectal carcinoma and HGSC GEMMs, respectively. As described in the gene-calling section, flox sites were made into a separate gene for the CN calling above, so we focused on the log_2_ CNR signal from the flox regions of *Brca1*, *Trp53*, *Rb1*, and *Nf1* for the HGSC samples and *Apc* and *Trp53* for colorectal carcinoma samples. Both cancer cohorts were filtered by requiring at least 30% tumor content which was calculated by subtracting the CNR from 1 using *Trp53* and *Apc* flox site CNRs for HGSC and colorectal carcinoma, respectively. The HGSC samples were used to compare the flox sites between the deletion sites for 40 of 51 (>30%) HGSC GEMMs sequenced by both MiMouse and our previously published HGSC panel (31). Log_2_ CNR values for the flox sites were compared between sequencing technologies using a correlation test. When using the sub-gene deletion data for colorectal carcinoma, we used them to analyze the biallelic inactivation of *Apc*. We first created a ratio of log_2_ CNR for the flox sites for *Trp53*:*Apc* as all samples only had one allele of *Apc* targeted for a deletion by the flox site. To test the biallelic inactivation of *Apc*, we leveraged three different genotypes present in our colorectal carcinoma mice with tumor content greater than 30%: AK, AKP ^R270H/flox^, and AKP^flox/flox^. These genotypes have expected ratio for *Apc:Trp53* log_2_ CNR signal for flox site inactivation of 0:1, 1:2, and 1:1, respectively. We then used the function glm from the stats package to model the relationship between the different genotypes (independent variable) and ratio of signal between the flox sites (dependent variable). The regression coefficients and their significance were then extracted to see whether biallelic inactivation of *Apc* could be observed through the different genotypes.

The fraction of genes altered (FGA) was calculated the same for every sample in which any gene with a |log_2_ CNR| > 0.2 was considered altered. Then the number of genes altered was divided by the total number of genes being measured. FGA was used in two different analyses in which we wanted to show (i) overestimation due to smaller panel sizes (32 vs. 119 genes) and (ii) comparison of genomic instability between our GEMMs and human tumors (using the same 119 genes on MiMouse). For FGA analysis, 119 of the 136 genes were chosen to be used as they were autosomal genes with more than three amplicons covering the gene. Although we aimed to have at least 10 amplicons (accounting for filtering by Ion Torrent), as shown in Supplementary Table S1, some genes did not have appropriate number of amplicons assigned during the design. The former analysis was done between the 40 HGSC GEMMs described previously (31), for which we calculated FGA using 32 and 119 genes from the previous HGSC-focused panel and MiMouse, respectively. The same set of 119 genes on MiMouse was used for the calculation and comparison of FGA between our GEMMs and human tumors from TCGA. Gene-level data were downloaded from cBioPortal for colorectal carcinoma and HGSC tumor, and FGA was calculated the same way as for the MiMouse sequenced samples. Like the previous analyses, samples were filtered for a minimum tumor content (>30%). The same gene-level data were used to calculate copy-level scores as a surrogate to measuring the homologous recombination deficiency (HRD) phenotype in our targeted panel. For each chr arm, we calculated the number of copy states i.e., gain, loss, or no change requiring at least two adjacent genes for a state to be present and then summing up the number of states for each sample. Plots and statistical tests for all gene-related analyses were performed in R using the ggplot2 library (74).

### Infinium array QC, aneuploidy calling, and validation

The Infinium Array (Illumina CCGP GIGA-MUGA-24 kit) was performed by the Advanced Genomics Core at the University of Michigan using 100 ng of rescued FFPE DNA. QC was run on the FPPE samples using the Infinium FFPE QC Kit (WG-321-1001) prior to rescuing the FFPE samples using the Illumina Infinium FFPE DNA Restore Kit.

As per instructions on the ASCAT (RRID: SCR_016868) github page, a custom GC correction file was created for the Infinium array using the createGCcontentFile.R script (80). The B-allele frequency parameters used for maxHomozygous, proportionHetero, proportionHomo, proportionOpen, and segmentLength were 0.05, 0.08, 0.83, 0.07, and 100, respectively. ASCAT was run using a range of tumor purities and ploidies. The purities were limited to within ±5% of the predicted tumor content from *Trp53* flox site log_2_ CNR found from MiMouse and a range of initial ploidies of 1.6 to 4.4. A limited range of tumor purities decrease the number of ASCAT purity and ploidy inference solutions. The companion R package for the array, argyle, was run on the raw Infinium array data for QC and marked the entire set of samples as lower quality, although as the authors state samples that are non-euploid can affect how well the R package gauges QC (83). The rest of the pipeline was run with its default parameters and custom R scripts were used to extract goodness-of-fit metrics and intermediate data points in order to manually curate the final purity and ploidy estimations for each sample. For each sample, we created CN plots of segments and B-allele frequencys in order to determine best the ploidy and purity solution. Gene-level copy number for comparison with MiMouse was called for the array by querying overlaps between the segments and the CGP bed file. All plots were made with ggplot2 in R (74).

Aneuploidy calls were made for the MGA data by taking the rounded integer level segments and applying the workflow described above using an *R*_*c*_ threshold of *R*_*1.0*_ ≥ 0.8. Only the 19 autosomes were considered for comparison as there are no short arms in mice and only autosomes were considered. Taken together with the aneuploidy calls previously described above for MiMouse, the aneuploidy statuses for both sequencing technologies were converted into factors (gain, loss, and no change) and used to calculate performance metrics based on PPA. Confusion matrices and performance metrics were calculated in R using the package caret (84). Graphs of these metrics were made with ggplot2.

Comparison of aneuploidy events in our MiMouse-profiled HGSC models with those from other studies was performed by visually determining aneuploidy events (chromosome level gain or loss = ±1) and sub-chromosome level gains/losses (±0.5) by visual evaluation of published genome-wide CN plots from all individual samples (*n* = 53) from seven published studies of HGSC models (85–91). Events per chromosome arm were summed across the 53 samples (both including and excluding sub-chromosome–level events). Aneuploidy events across the 113 evaluable HGSC GEMMs profiled by MiMouse were scored and summed per chromosome for comparison.

### Comparison of genotyping by Infinium array and MiMouse

Samples sequenced using MiMouse had their genotyping SNPs force-called as variants using a docker container for tvc (5.18.1). The custom docker container also used bcftools (1.3.1) and was used to filter the tvc output (71). Integrative Genomics Viewer was used to manually inspect positions that were consistently called as indels between all sequencing runs (92). Integrative Genomics Viewer was used to look for adjacent nearby homopolymer and strand biases that have been known to be confounding factors in Ion Torrent–based sequenced (21). PLINK (1.90b6.9; RRID: SCR_001757) was run with a missing genotype parameter of 0.2 to create the fam, bed, and ped files (93). A custom pop file was created using reference samples of each mouse strain created from aligning mouse strains of interest fastq files from Mouse Genotyping Project with mm10 and force-calling the positions of interest with mpileup from samtools (71, 94). These files were used in conjunction to run ADMIXTURE using a k of 5, which is the parameter for the expected number of reference populations. Because estimating the admixture of founder strains via ADMIXTURE is dependent on the number of SNPs used, the number of reference populations and the divergence of the strains were used. Validation was performed below using an extensive haplotype reference in order to determine factors like noise thresholds (73). Genotyping of Infinium samples was done using Charles River and the Jackson Laboratory’s HaploQA (95). The Infinium sample map files and array signal files were uploaded to the HaploQA site. The predicted haplotypes were concatenated and summed for each strain for comparison with admixture inference. Statistical tests of the mouse strain estimations between the two technologies like calculating residuals and the creation of structure plots were done using the R packages stats and ggplot2.

### Cross-species analysis of aneuploidy events

Calculations of the colorectal carcinoma–specific events were done using TCGA data downloaded from cBioPortal (57). Results from GISTIC2.0’s broad analysis were used as input for the aneuploidy status of a chr arm (autosomal; ref. 96). For each chr arm in colorectal carcinoma, a Fisher exact test was run in a pairwise fashion to compare colorectal carcinoma with all other solid tumors sequenced in TCGA. FDR-corrected *q* values were then calculated to control for multiple testing. Pairwise occurrence was then calculated for chr arms found more frequently altered in colorectal carcinoma than at least half of the other cancers. Then a Fisher exact with FDR correction was ran for the resulting co-occurring chr arm comparison. The −log_10_ (*q* values) were then visualized as boxplots by chr arm change.

Because of the low number of samples in our colorectal carcinoma GEMMs, aneuploidies were tested for significant occurrence by using a permutation test. Briefly, a matrix of sample by chr arm with aneuploidy status as the value was resampled without replacement within each sample. A two-sided z-proportional test statistic was calculated for both proportion of arm gain and loss (separately) for a specific autosomal arm compared with the 18 other arms in our mice. This process was run 10, 000 times, and *P* values for the occurrence of gain or loss of each arm were calculated from our observed frequencies compared with the background (permutation). A similar resampling was run for human colorectal carcinoma with a matrix of samples by chr arms, excluding acrocentric chrs, for comparison of syntenic blocks.

Based on our three hypotheses for the importance of aneuploidies in a cross-species analyses, we calculated the log OR for gains of colorectal carcinoma–specific chr arms as described above and their syntenic mouse chromosomes. Briefly, synteny maps were created with SynBuilder at its highest resolution of 150 kb between hg19 and mm10 (97). The log OR (gain of chr arms of interest in human and mouse) was then calculated for the three different scenarios (all syntenic, singular arm gains, or no arm gains) for our observed data and for each of the 10,000 matrices of resampled data from the permutation test done on mouse and human colorectal carcinoma. *P* values were then calculated like our permutation tests based on the log OR from the resampled matrices and our observed logOR. Circos plots and percent synteny were calculated using the synteny map from SynBuilder and the circlirize (RRID: SCR_021126) R package (98). The syntenic areas found significant were overlapped with cytobands found to be significantly altered through GISTIC analyses for colorectal carcinoma using GenomicRanges (75). Gene dependency scores that were uncorrected for copy number were downloaded from the DepMap website (depmap). Expression and meta data for the cell lines were obtained using the R package depmap, whereas the aneuploidy status was obtained from a previous study analyzing the same sequenced cell lines from CCLE (99, 100). Only dependency scores from the CRISPR knockout assays were used for analysis.

## Results

### MiMouse CGP considerations and development: selection of target genes

**Figure 1.**
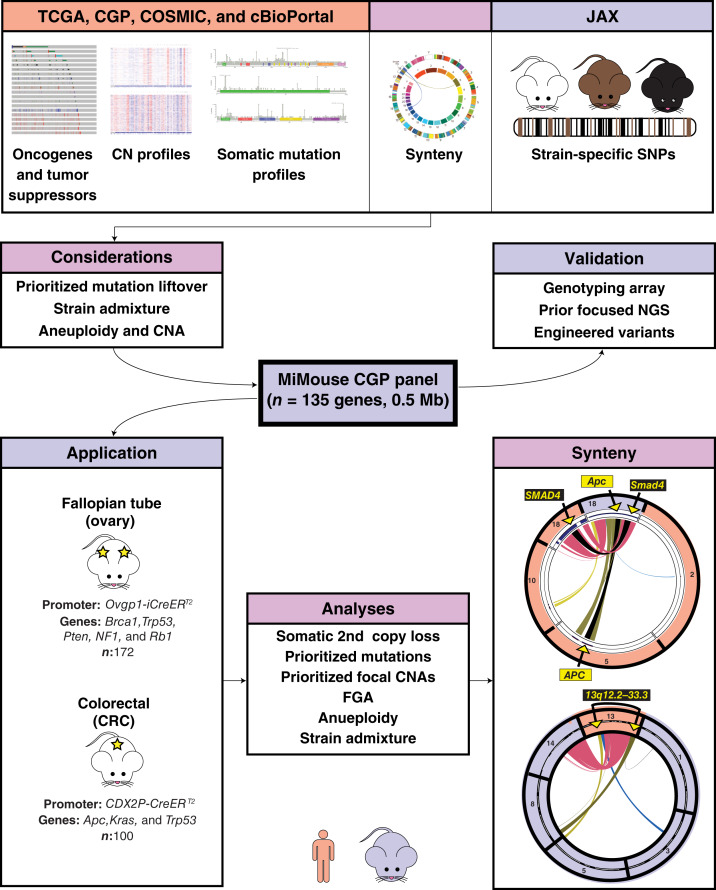
Overview of the development and application of MiMouse for mouse CGP and genomic credentialing. Human (peach)- and mouse (lavender)-specific resources were considered through simulations to develop MiMouse, a 135-gene (0.5 Mb) CGP assay designed to be compatible with minute GEMM tumor lesions from all stages of neoplasia. Validation for various alteration and genomic signature classes was performed using a combination of orthogonal technologies. We applied MiMouse to a large cohort of tumors from fallopian tube (ovary) and colorectal (colorectal carcinoma) models; see Fig. 4 for detailed genotypes and histology. In addition to standard CGP profiling and demonstration of utility for genomic credentialing (e.g., comparison of FGA between human and mouse ovary vs. colorectal carcinoma tumors), our study of aneuploidy in colorectal carcinoma tumors highlighted the importance of synteny. For example, two key tumor suppressors, *APC*/*Apc* and *SMAD4*/*Smad4*, show unique arrangement in humans (on separate chrs) and mice (both on chr 18). Likewise, in humans, three peaks (spanning from 13q12.2-33.3) have been identified in the recurrent, highly colorectal carcinoma–specific 13q gain; this region maps to five distinct mouse chrs. Images of human-specific resources were from cBioPortal (57); the synteny resource image was from Jax synteny browser (126). CRC, colorectal carcinoma.

To begin to address the unmet need of developing an FFPE-compatible mouse CGP approach that is both applicable to all stages of neoplasia in mouse tumor models (most commonly GEMMs) and capable of credentialing the full spectrum of human tumor genomic alterations, herein, we systematically assessed considerations in cross-species CGP assay construction, as well as developed and applied a 4,262-amplicon (0.5 Mb) multiplex-PCR–based panel (MiMouse) to >250 mouse tumor samples arising in high-fidelity HGSC and colorectal carcinoma models. Highlighting unique cross-species considerations, we focus here on describing the issues required to prioritize somatic mutations, strain composition, and aneuploidy. Importantly, the MiMouse panel represents one way to balance applicability/throughput versus suitability for comprehensive genomic fidelity; however, the considerations are broadly applicable to any genomic study of GEMMs (Fig. 1).

Extensive efforts to define recurrently altered (mutations or CNAs) somatic driver genes across nearly the entire spectrum of human tumors have been described (8, 66, 101, 102), with CGP panels targeting 100s of such genes having been used to profile hundreds of thousands of human FFPE tumors (13, 21, 33, 60). Likewise, the genomic footprint of such panels needed to estimate clinically relevant genomic signatures, such as that required to generate a point estimate for tumor mutation burden (TMB) at the clinically relevant threshold of ≥10 mutations (Muts)/Mb, has also been extensively reviewed (103). As the considerations for translating these considerations to mouse-specific CGP panels are similar, they were not explored further herein. With regard to the initial MiMouse target gene set, we balanced pan-tumor/genomic alteration class applicability versus cost/the initial focus on HGSC and colorectal carcinoma to target 135 genes, including 30 TSGs, 31 oncogenes, and 74 additional cancer-related genes (Supplementary Table S1). Desired amplicon coverage was based on previously described approaches to enable detection of hotspot mutations and amplification assessment of oncogenes, full CDS coverage of TSG, and focal CNAs in cancer-related genes (38).

### MiMouse CGP considerations and development: cross-species interpretation of somatic mutations

Designing amplicons to target human hotspot (recurrent and therefore considered driving/prioritized) mutations in the 31 MiMouse oncogenes required appropriate liftover of hotspot mutations to the mouse genome, both for panel design as well as evaluation of the functional significance of somatic mutations in mouse tumors. As this has not been comprehensively addressed previously, we systematically evaluated this aspect of mouse CGP through considering nonsynonymous mutations in 270 total TSGs, oncogenes, and cancer-related genes from the Memorial Sloan Kettering Cancer Center institutional effort performing CGP on ∼10,000 advanced solid tumor samples contained within OncoKB, as reported by Zehir and colleagues (Supplementary Fig. S1A; ref. 60).

From our cross-species mapping, 87% (3,613 of 4,155) of the individual aa positions in the 270 genes were found to be shared hotspots [corresponding aa and positive local conservation score (see “Materials and Methods”)] between humans and mice. We explored the association of several factors with cross-species hotspot conservation, including local conservation score, mutation frequency in the OncoKB cohort, whether a mutation resides in an oncogene or TSG, and location in/out of protein domains. As expected, the local conservation score (continuous) of a mutation had the most significant effect on the hotspot being shared (OR = 2.31 per increase of 1 unit of conservation score, *P* < 0.001). Notably, frequency in the OncoKB cohort, residing in a protein domain, and stop–gain mutations (vs. missense mutations) were significantly associated with a hotspot being shared between species, independent of the local conservation score (Supplementary Fig. S1B); when not accounting for the local conservation score, all variables were significant, with a hotspot residing inside versus outside a domain having the largest significant effect (OR = 3.98; *P* < 0.001) on conservation (Supplementary Fig. S1C). Although not surprising, these findings refine our understanding of driver/prioritized (by OncoKB) mutations in humans, with most recurrently affected aa positions in humans being evolutionarily conserved in mice and occurring in functional domains. In addition, although the entire CDS of TSGs are typically targeted by CGP panels, these results can help inform the design of coverage strategies for oncogenes (in which only recurrent hotspots may be targeted along with sufficient amplicons for CNA detection).

### MiMouse CGP considerations and development: aneuploidy and CNA detection considerations

**Figure 2.**
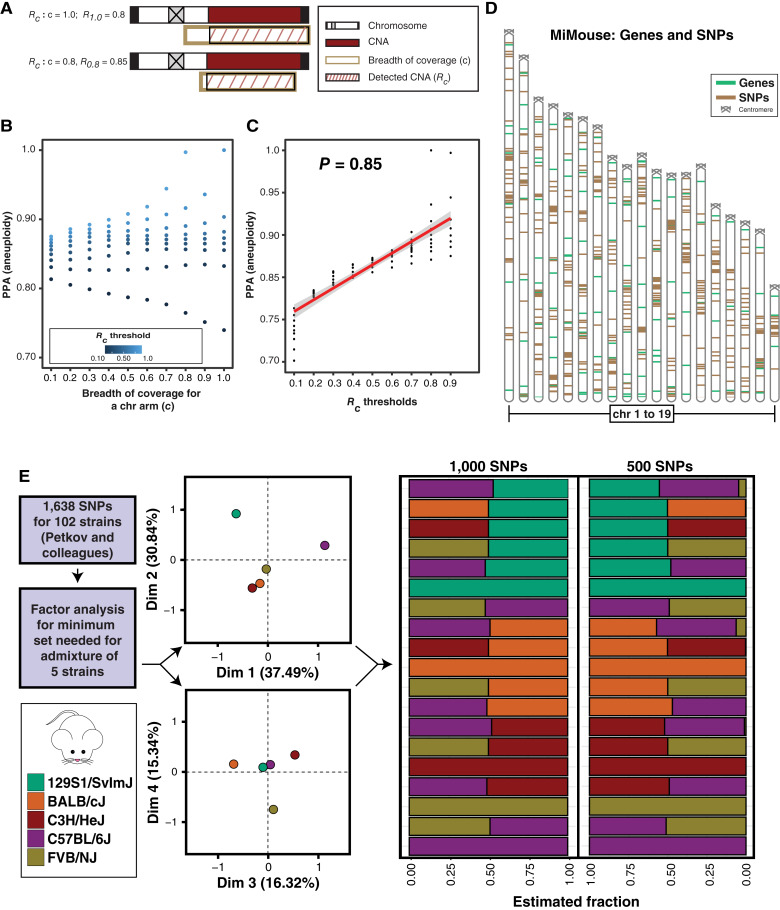
Aneuploidy and admixture detection considerations for mouse CGP by MiMouse. **A,** To simulate the effects of reduced coverage by CGP vs. near genome-wide technologies, we developed a metric, *R*_*c*_, in which *c* is the breadth of coverage for a chr arm (gold) centered at the midpoint of the chr arm, and *R*_*c*_ is the proportion of the chr arm altered (hatched maroon region) by a CNA (maroon) specific to *c*. The resulting *R*_*c*_ for the same CNA measured by assays with different *c* (*R*_*1.0*_ = near-genome wide) is shown. Centromeres are indicated by an X. **B,** Dotplot of PPA for aneuploidy detection across human tumors (*n* = 6,591 diploid tumors from TCGA) by *R*_*c*_ thresholds for detecting aneuploidy, with variable *c* (*x*-axis) and *R*_*c*_ thresholds (color gradient per scale). **C,** Pearson correlation (*r*) between varying *R*_*c*_ thresholds (*x*-axis) and PPA of aneuploidy detection for autosomal aneuploidy arms (TCGA data). **D,** Ideogram [*Mus musculus* assembly GRCm38 (mm10)] of autosomal MiMouse genic targets (green) and SNPs (gold). Centrosomes are indicated. **E,** To determine a minimal set of SNPs capable of distinguishing five common mouse strains (see legend) from the 1,638 identified by Petkov and colleagues (as discriminatory of more than 100 strains; ref. 69), we performed factor analysis. Biplots (left) of the dimensions from the factor analysis (factor scores plotted) are shown, along with structure plots (right) showing *in silico* pairwise crossing of the mouse strains of interest and the resulting ADMIXTURE strain estimations (mean) using reduced sets of 1,000 and 500 SNPs from factor loadings.

Although both comprehensive aneuploidy and focal CNA profiling are routinely done by SNP arrays, WES, and/or WGS, given the limited direct therapeutic relevance of most low-level CNAs, considerations for CGP construction to maximize aneuploidy detection from minimized panels have been less well described (although both hybrid capture and multiplex PCR approaches have been clinically validated for amplification and homozygous deletion detection; refs. 32, 39, 104). As described in detail in the Supplementary Results and Figs. S2–S4, through comprehensive simulation using human TCGA tumors and CCLE samples, we demonstrate that simulated CGP aneuploidy detection performance [as measured by PPA versus approaches with effectively genome-wide coverage (e.g., array CGH or WES)] is affected by each chr arm’s breath of coverage (*c*) and marker density. As shown in Fig. 2A–C, we determined that a metric *R*_c_, the coverage-specific proportion of the chr arm altered (Fig. 2A), when used as a threshold for calling aneuploidies (regardless of *c*) was directly correlated (Spearman rank correlation = 0.85) with higher PPA (0.75–1.0) when compared with reference calls made with an *R*_*1.0*_ threshold ≥ 0.80 [Fig. 2B and C; see Supplementary Results for reference calling approach assuming near genome-wide coverage (*c* = 1.0)]. As expected, performance (PPA) was lower in simulations with sparser markers (independent of *c*); however, false-positive rates could be reduced by requiring a proportion of the genes on the arm to be altered in the same direction (directionality filter; see Supplementary Results). Based on our simulation, the 135 gene targets in the final MiMouse panel enabled a minimum *c* = 0.84 per chr arm (range, 0.84–0.96). Inclusion of additional amplicons targeting mouse SNPs meant for admixture inference improved overall marker density (Fig. 2D; Supplementary Fig. S4).

### MiMouse CGP considerations and development—admixture detection considerations

As described above, although genetic ancestry is increasingly being considered in evaluating the distribution of recurrently altered somatic alterations in human tumors (105–107), the use of inbred mice and impact of strain admixture on GEMM phenotypes support careful consideration in the genomic credentialing of such modeling. Although comprehensive admixture can be definitively assessed using large amounts of non-tumor DNA (which is not routinely limited) by genotyping arrays with >100,000 markers (95), a previous phylogenetic study showed that a set of 1,638 SNPs could distinguish more than 102 laboratory and wild mouse strains (69), representing a potential upper bound on the number of amplicons needed to be added to a mouse CGP panel to enable tumor-only admixture evaluation. Hence, we first sought to evaluate the minimum number of these 1,638 SNPs (all were intergenic and had no overlap with our gene targets) required to detect different strain admixtures in five commonly used strains (C57BL/6J, BALB/cJ, 129S1/SvmJ, FVB/NJ, and C3H/HeJ; ref. 69) using factor analysis. As shown in Fig. 2D, four loadings obtained from factor analysis were enough to differentiate the strains, with loadings one and two differentiating 129S1/SvImJ from C57BL/6J and the three other mouse strains, and loadings three and four differentiating each of the three remaining mouse strains (BALB/cJ, FVB/NJ, and C3H/HeJ). Although similar analysis on sets of 1,000 and 500 SNPs resulted in increased error (root mean square errors of 1.2% and 17%, respectively), we added the 500 SNP set to MiMouse to minimize panel size (which should still be sufficient to infer mouse strain with admixtures >17%; Fig. 2D). The addition of these SNPs was also predicted to improve MiMouse’s ability to call aneuploidy by increasing the overall marker density from a range of one marker per 23 kb to 56 Mb window (median 15Mb) to one marker per 23 kb to 26 Mb window (median, 1.6 Mb; Supplementary Fig. S4).

### MiMouse CGP considerations and development—summary and validation

Given the above considerations, in total, the MiMouse panel used 3,772 amplicons to cover 135 genic targets, consisting of 533 amplicons for hotspot mutation and CNA detection in 31 oncogenes, 2,358 amplicons covering the entire targetable CDS of the 30 TSGs, and 881 amplicons targeting 74 cancer-related genes for CN detection. Combined with the 490 amplicons covering genotyping SNPs (10 of 500 submitted failed AmpliSeq amplicon designing), the MiMouse panel totals 4,262 amplicons covering 0.5 Mb (Fig. 1). Importantly, although ∼0.7 to 0.8 Mb of genomic footprint is required to generate a precise point estimate for TMB at the clinically relevant threshold of ≥10 Muts/Mb (103), given that assessing TMB was not a primary application of initial interest, we chose to proceed with the development of the 0.5 Mb MiMouse panel.

**Figure 3.**
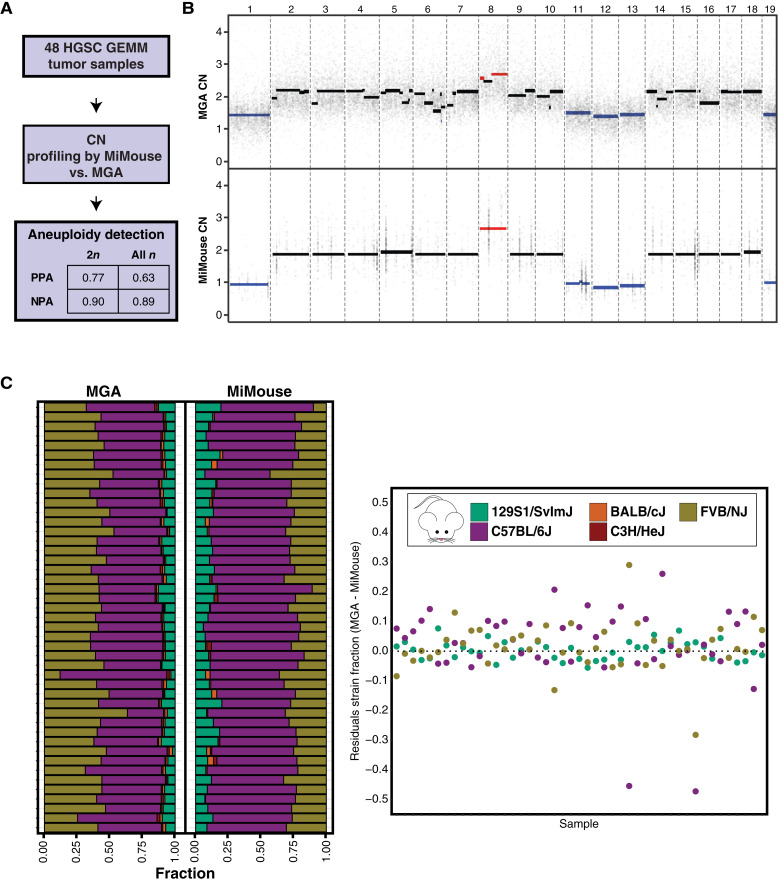
Validation of aneuploidy and admixture detection for mouse CGP by MiMouse. **A,** For validation of aneuploidy detection by MiMouse, 48 HGSC FFPE tumor samples were tested by MiMouse and Infinium MGA. PPA and negative percent agreement (NPA; MGA as reference) are shown. **B,** Probe/amplicon (dots) based unrounded CN calls (scaled for purity and ploidy from ASCAT) for a representative HGSC tumor sample using MGA (from ASCAT; top) and MiMouse (our pipeline; bottom), with the CN status colored by each assay’s final pipeline output as no change (black), gain (red), and loss (blue). **C,** Strain composition structure plots (left) of the same HGSC samples (45 of 48 passing genotyping QC by MGA and MiMouse) from MGA (HaploQA) and MiMouse (ADMIXTURE). Residuals (right) between strain estimations per sample are plotted [overestimation (negative on *y*-axis) and underestimation (positive on *y*-axis) by MiMouse, respectively]. Strains are indicated by the legend on right.

To validate the critical assumptions made in aneuploidy and admixture detection features of the MiMouse panel, we performed MGA (Infinium) on aliquots of the same FFPE-isolated DNA from 48 HGSC mouse tumor samples with >50% estimated tumor content (by MiMouse estimation). Although available earlier than our colorectal carcinoma models, by using HGSC tumor samples, we expected a lower PPA compared with our aneuploidy detection simulation, as performance varies based on human tumor type (Supplementary Fig. S3), in part due to the large number of both aneuploidy and non-arm–level copy-number events in HGSC (characteristic of HRD; refs. 53–55, 108, 109). As expected, arm-level calls made with any *R*_*c*_ threshold without the directionality filter performed poorly with a high false-positive rate. For example, using an *R*_*0.6*_ ≥0.8 produced a PPA of 0.35 for diploid samples and 0.40 for polyploid samples (Supplementary Fig. S5). However, with incorporation of the directionality filter, as shown in Fig. 3A, using a *R*_*0.6*_ threshold ≥0.8, the MiMouse panel (vs. MGA) produced PPA of 0.77 and 0.63 for diploid (*n* = 35) and all samples (*n* = 48; including 13 polyploid samples by manual inspection of ASCAT profiles), respectively, across the 62 autosomal chr-level gains and losses detected by MGA. As expected, PPA for aneuploidy detection by MiMouse (0.7–0.8 irrespective of *c* or *R*_*c*_ threshold used) fell within the simulated range for HGSC (0.6–1.0; Supplementary Fig. S3). Example copy-number plots for the same sample profiled with both MGA and MiMouse are shown in Fig. 3B, and the correlation of autosomal gene-level CN calls [median Pearson correlation = 0.75; 95% confidence interval (CI), 0.67–0.77] is shown in Supplementary Fig. S6.

When evaluating mouse strain estimation, using ADMIXTURE and HaploQA for genotyping analysis from MiMouse and MGA, respectively, 45 of 48 (94%) samples sequenced using both technologies provided usable results to compare mouse strain estimations [the other three samples did not meet the minimum genotyping rate (>80%) from MiMouse and/or MGA]. Likewise, we used a conservative threshold of >5% to consider a strain detected by MGA (and therefore MiMouse) based on genotyping imputation error rates of inbred mice (110). Using this threshold, by MGA, all validation cohort samples consisted of C57BL/6J, 129S1/SvImJ, and FVB/NJ, with 0.98 PPA at the strain level by MiMouse (Fig. 3C), with residual standard errors for the strains C57BL/6J, FVB/NJ, and 129S1/SvImJ of 0.13, 0.08, and 0.03, respectively (Fig. 3D). Unexpectedly, we observed that ADMIXTURE ran on MiMouse-sequenced samples utilized a smaller set of genotyping SNPs (range, 362–411 of the total 490 SNPs) because of challenges in variant calling in homopolymer regions (Supplementary Fig. S7), a well-described Ion Torrent error profile (39), supporting consideration of this artifact in SNP selection and/or advanced background correction approaches (111). Nevertheless, in addition to high PPA, genotyping by MiMouse also showed reasonable performance for strain proportion within a mouse, i.e., C57BL/6J > FVB/NJ > 129S1/SvImJ (Kendall coefficient of agreement of 0.76). Taken together, using MGA as an orthogonal technology, these results support the analytic methods used herein for MiMouse, confirm important considerations in the design of a mouse CGP panel for aneuploidy event and admixture characterization (such as marker spread for aneuploidy event detection and sequencing platform–specific limitations), and highlight tradeoffs made to minimize the panel size for the initial MiMouse versus larger CGP panel designs.

Lastly, although we have extensively validated the ability of similar human amplicon-based CGP panels to accurately detect focal amplifications (CN ≥ 6) and homozygous deletions (including sub-gene deletions; CN = 0) using established pipelines (38), we next evaluated 51 FFPE MiMouse-profiled HGSC samples that were previously evaluated (from replicate DNA isolate aliquots) by our HGSC-focused panel (31). Although no focal amplifications were detected by either panel in these 51 samples, we could evaluate the concordance of sub-gene deep deletion calling from Cre-flox sites within *Brca1*, *Trp53*, *Rb1*, and *Nf1* (with known/expected status based on the strain for each sample). As shown in Supplementary Fig. S8, across the 40 of 51 samples with sufficient tumor content (>30%) to be informative for this analysis, the pairwise log_2_ CNR estimates were highly correlated for these four loci (Pearson *r* = 0.84–0.93). Likewise, although no somatic mutations were detected by either panel in these 51 samples, as shown in Supplementary Table S2, we confirmed that across evaluable MiMouse-sequenced colorectal carcinoma mouse tumor samples (see below) with introduced human mutations (*BRAF* p.V600E and *TP53* p.R270H), these human mutations were only detected in the nine of nine samples with introduced *BRAF* p.V600E mutations and the 21 of 22 of samples with *TP53* p.R270H mutation, respectively (the sample without the expected mutation minimally passed our usual uniformity QC metric).

### MiMouse CGP application to GEMMs

**Figure 4.**
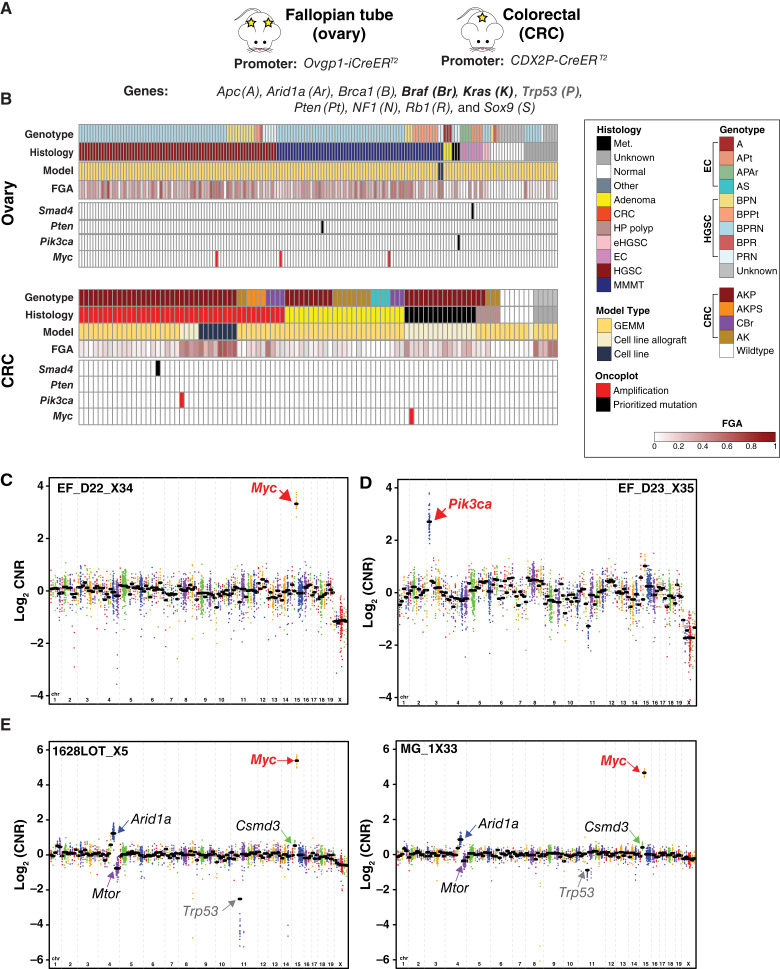
MiMouse for CGP and genomic credentialing across the neoplastic spectrum of two large cohorts of inducible fallopian tube (ovary) and colorectal (colorectal carcinoma) tumors. **A,** Promoters and transgenes used in the cohorts. Ovary and colorectal carcinoma tumor cohorts tested by MiMouse. Genes in bold were activated, and those in regular were inactivated; *Trp53* (bold gray) was either inactivated through deletion or a point mutation was expressed (see main text for specific models). Abbreviations for genotypes (as used in the heatmap) are indicated. **B,** MiMouse cohort characteristics and CGP results from the ovary (predominantly HGSC; top, total *n* = 172) and colorectal carcinoma (bottom, total *n* = 100) cohorts are shown. Genotype, histology (CRC, carcinoma; eHGSC, early HGSC; HP, hyperplastic; Met., metastasis; MMMT, carcinosarcoma) and model type (GEMM, GEMM tumor tissue) are shown. Gene-level FGA (see main text) is shown according to the color scale (gray indicates no value due to QC failure, see Supplementary Fig. S9). All detected somatic, prioritized, amplifications (red), deep (homozygous) deletions (blue; none detected), and prioritized somatic mutations (black) across the cohorts are shown. **C** and **D,** CN plots of two colorectal carcinoma samples with focal amplifications in *Myc* or *Pik3ca* (indicated by arrows) are shown. The log_2_ CNR (vs. pseudo-normal) of individual amplicons are plotted, with genes (different colors) in genome order (chrs on bottom of plot) and gene-level log_2_ CNR indicated by black bars (black bars). **E,** CN plots of two histologically distinct HGSC tumors arising from both ovaries of the same mouse (1628LOT_X5 = left; MG1_X33 = right). Shared *Myc* amplifications are indicated. The remaining somatic CN profile (including gains in *Arid1a* and *Csmd3* and loss of *Mtor*) is consistent with clonal origin [*Trp53* (gray), which includes the engineered flox amplicons lost after induction and is shown for estimation of relative tumor content]. CRC, colorectal carcinoma.

As described above, we initiated the MiMouse project to develop a CGP panel with broad applicability for genomic credentialing of mouse tumor models. Hence, we next applied MiMouse CGP testing to a large cohort of tumor samples from HGSC [and additional fallopian tube (ovarian) endometroid carcinoma (EC) models] and colorectal carcinoma tumor models processed by multiple fixation methods (Fig. 4A). This cohort included multiple sample types, including mouse tumor cell lines, syngeneic implant models, and FFPE lesions representing the full neoplastic spectrum (precursor lesions to metastases) from GEMMs. Importantly, this cohort allowed us to assess the applicability of the MiMouse panel for aspects of genomic credentialing using the above-described pipelines and approaches; importantly, the inclusion of a subset of samples previously assessed by our smaller HGSC-focused panel (as described above; ref. 31) also enabled us to directly evaluate the importance of increased resolution for assessing relevant genomic features such as genomic instability.

As shown in Fig. 4B, across the 272 total mouse samples assessed by MiMouse from the HGSC (*n* = 172) and colorectal carcinoma cohorts (*n* = 100), 258 [94.8%; 226 neoplastic samples, 16 normal samples, and 16 unknown tumor (histology not available)] samples yielded evaluable CGP results using our previously established QC metrics (38, 39). As shown in Supplementary Fig. S9 and Table S3, across these 258 samples that passed sequencing QC measurements, the median coverage depth, on-target read percentage, and uniformity were 437x (range, 106x–1,485x), 99% (range, 86%–99%), and 97% (range, 81%–99%), respectively. A total of 254 of the 258 (98%) samples could be genotyped by MiMouse [the remaining samples did not meet the minimum genotyping rate (>80%)]. Consistent with the results in the 48-sample HGSC cohort tested by MiMouse and MGA, as shown in Supplementary Fig. S9, all evaluable MiMouse-sequenced HGSC samples (*n* = 156) were mixtures of C57BL/6J (median, 62%), FVB/NJ (median, 25%), and 129S1/SvImJ (median, 11%), whereas evaluable colorectal carcinoma samples (*n* = 97) were either mixtures (*n* = 74) of C57BL/6J (median, 72%) and 129S1/SvImJ (median, 27%) or mixtures (*n* = 23) of C57BL/6J (median, 70%) and 129S1/SvImJ (median, 16%) and FVB/NJ (median, 9%); results were expected based on GEMM construction (Supplementary Table S3).

Next, similar to standard clinical human CGP analysis, we assessed all evaluable MiMouse-profiled samples for prioritized mutations and high-level CNA [amplifications (CN ≥ 6) or homozygous deletions (CN = 0)] in the 135 oncogenes, TSGs, and cancer-related genes targeted by MiMouse using established amplicon-based mutation and CNA calling approaches (38, 39) supplemented with the LiftOver database described above for interpreting the significance of somatic mutations. Across the 242 evaluable tumor samples, in addition to two synonymous mutations, we identified a total of 13 nonsynonymous mutations [variant allele frequency (VAF); range 10%–55%], consisting of 11 nonsynonymous single-nucleotide variants and two stop-gain mutations (Supplementary Table S4). As shown in Fig. 4B, a *Pten* p.335X stop gain mutation at 14% VAF in an HGSC GEMMs was the only mutation directly prioritized as functionally significant; however, three additional GEMM samples (one each HGSC, EC, and colorectal carcinoma) each harbored a single prioritized mutation, representing the human equivalent of *PIK3CA* p.E545K (HGSOC; *Pik3ca* p.E545K, 55% VAF), *SMAD4* p.R361G (colorectal carcinoma; *Smad4* p.R360G; 19% VAF), and *SMAD4* p.E526Q (EC; *Smad4* p.E525Q 10% VAF; Supplementary Table S5).

Although no prioritized homozygous deletions were detected in any of the 242 evaluable tumor samples (outside of floxed targets used in model creation; see above), we observed a total of five high-level amplifications: a *Myc* amplification in a colorectal carcinoma GEMM, a *Pik3ca* amplification (log_2_ CNR of 2.7) in a colorectal carcinoma GEMM, and three HGSC with *Myc* amplifications (Fig. 4C–E; Supplementary Table S5). Of note, two Myc amplifications occurred in distinct HGSC (from each ovary) from the same mouse, with the overall CN profile of these bilateral HGSC tumors confirming clonality rather than independent tumors arising in each ovary (Fig. 4E). CN data for all profiled tumors are available in Supplementary Table S6. Taken together, these results highlight the ability of the MiMouse panel to detect both prioritized mutations and high-level CNAs, demonstrate the need (at least in these models) to profile many tumors to identify non-engineered somatic driver mutations/focal CNAs (likely due to the substantial engineered drivers present), and notably highlight the utility of our LiftOver approach for prioritizing mouse missense mutations.

### MiMouse panel for genomic credentialing of GEMMs: HGSC models focusing on FGA

Beyond expanding the number of genes for which prioritized mutations and high-level CNAs could be detected (beyond our HGSC-focused panel), the primary motivation for developing the MiMouse panel was to enable more comprehensive genomic credentialing of neoplastic lesions (from precursors to metastases) from FFPE mouse models, including assessment of strain admixture, aneuploidy (including genome-wide patterns), and genomic instability (by FGA and by CN gains or losses). Hence, given that high genomic instability is a defining feature of human HGSC, including both aneuploidy (arm/chr level; Supplementary Fig. S10) and shorter alterations that are characteristic of an HRD phenotype, we first focused on using MiMouse to credential genome instability in our three previously characterized HGSC GEMMs that differ by the combination of engineered alterations: *Brca1*, *Trp53*, *Rb1*, *Nf1* (BPRN), *Brca1*, *Trp53*, and *Nf1* (BPN) and *Brca1*, *Trp53*, *Pten* (BPPt). A total of 117 of 157 (75%) HGSC tumor samples (three precursors, 113 primary tumors, and one metastasis) were of these genotypes and passed our minimum threshold of expected tumor content of 30%. Of the 40 samples that were excluded, 24% (38 of 157) of samples were of genotype or histology not of interest (including normal samples), and 1% (two of 157) of samples failed our minimum tumor content requirement.

Given the design considerations for the MiMouse panel (as above), rather than fully develop an HRD analysis pipeline that requires equal coverage across the genome, we focused on the ability of MiMouse to characterize FGA (at the gene level), including comparison with results obtained from these same models with our previous HGSC-focused panel, and in particular comparison of FGA between HGSC and colorectal carcinoma models. As shown in Supplementary Fig. S11A, although FGA estimates were correlated between MiMouse- and the HGSC-focused panel [Pearson *r* = 0.64 (95% CI, 0.41–0.78), *P* = 7.5e−6], estimated FGA was consistently higher by the HGSC-focused panel (median 0.53 vs. 0.43); given our calculated FGA from reduced gene sets using human TCGA HGSC data (Supplementary Fig. S12), these results agree with overestimation of genomic instability by the HGSC-focused panel, supporting the benefit of a larger panel for genomic credentialing.

**Figure 5.**
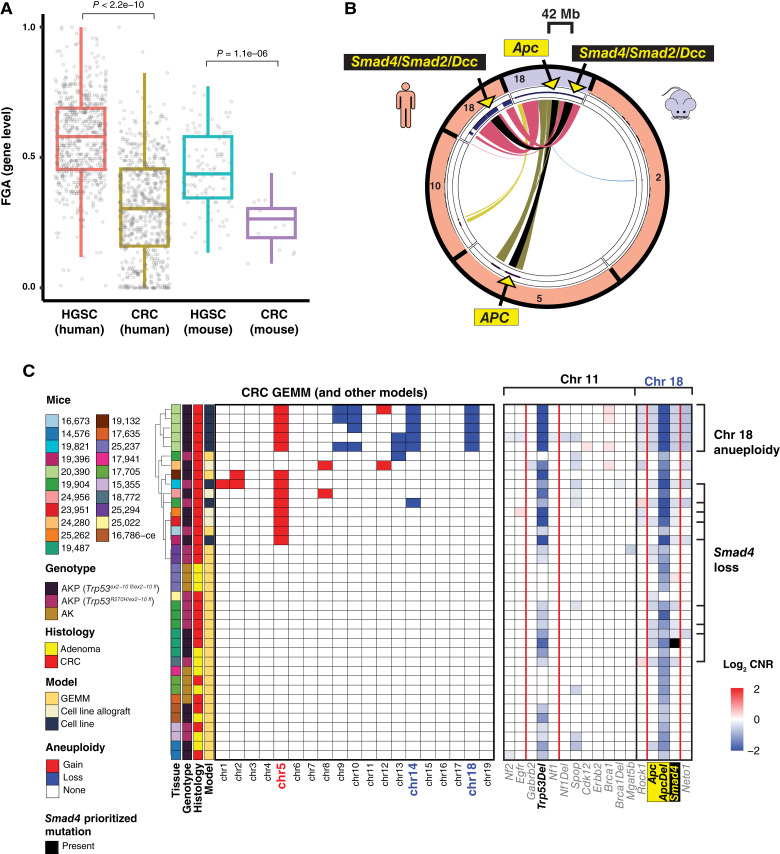
Genomic credentialing of increased FGA in fallopian tube HGSC and mouse genome arrangement–specific mechanisms of *Smad4* loss in colorectal carcinoma by MiMouse. **A,** Comparison of gene-level FGA was performed between human (TCGA) HGSC and colorectal carcinoma (*n* = 572 and 592, respectively) and MiMouse-profiled GEMM HGSC and colorectal carcinoma tissues (*n* = 113 and 16, respectively) using the evaluable MiMouse autosomal gene set (*n* = 119); FGA from individual samples are plotted with box plots shown (*P* values from comparison of distributions by Wilcoxon rank-sum tests are shown). **B,***APC* and *SMAD4*, two of the major tumor suppressors in colorectal carcinoma, show distinct genomic arrangement in humans vs. mice. Circos plot showing the syntenic mapping of *APC*/*Apc* and *SMAD4*/*Smad4* in humans (peach) and mice (lavender). Syntenic linkages are colored by the different mappings of mouse chr 18, which harbors both genes in mice, to all syntenic human chromosomes; linkages to *APC* (human chr 5) and *SMAD4* (human chr 18) are in black. **C,** Heatmap of aneuploidy by MiMouse for all evaluable colorectal carcinoma (10 adenomas and 28 carcinomas) samples is shown (red = gain; blue = loss). Individual mice, genotype, histology, and model type are indicated. Chromosomes found to be significantly gained are indicated in red; those significantly lost are in blue. The heatmap on right (chr11 and chr18) shows gene-level CN status (log_2_ CNR) according to the color scale, with the engineered *Trp53* floxxed alleles (*Trp53Del*), the floxxed (Del) and unfloxxed *Apc* alleles, and *Smad4* highlighted. Samples with *Smad4* disruption through chr 18 aneuploidy (*n* = 5), focal *Smad4* copy loss (*n* = 9), or prioritized *Smad4* mutation (*n* = 1; black) are indicated. CRC, colorectal carcinoma.

Next, as shown in Fig. 5A, consistent with human tumors [in which HGSC has significantly greater gene-level FGA than colorectal carcinoma (including when limited to MiMouse orthologues)], we confirmed that our HGSC GEMMs [median FGA 0.44 (95% CI, 0.41–0.48)] also had significantly greater FGA than our colorectal adenocarcinoma GEMMs [median FGA 0.26 (95% CI, 0.14–0.30); Wilcoxon rank-sum test *P* = 1.1e−6]. Interestingly, we also observed significant (by pairwise Wilcoxon rank-sum tests) differences in FGA within HGSC GEMMs based on their initial gene knockouts, BPPt having less FGA [median 0.33 (95% CI, 0.24–0.40)] versus BPRN [median 0.44 (95% CI, 0.39–0.48)] and BPN [median 0.58 (95% CI, 0.43–0.61); Supplementary Fig. S11B]. Of note, BPPt mice have significantly shorter latency after transgene induction to HGSC development versus BPRN, BPR, and BPN mice (∼32 weeks vs. >1 year; refs. 31, 56).

In addition to general measures of gene-level FGA, we also considered evaluation of patterns of genomic instability found specifically in HRD tumors, including sub-telomeric allelic imbalances or large-scale transitions, which should also differ in occurrence between human HGSC and colorectal carcinoma (55). Although evaluable by larger CGP panels with more evenly distributed genome-wide markers (20, 108, 112, 113), and consistent with the visibly different patterns of CNAs in HGSC and colorectal carcinoma GEMMs with high gene-level FGA (Supplementary Fig. S11C), we assessed whether a simple metric of summing the different CN states (gain, loss, or no change) per chr within a sample, which we refer to as FGA (copy-state score), could approximate a more complex FGA/HRD-like metric. In support, we found that HGSC GEMMs had significantly higher FGA (by copy-state score) versus colorectal carcinoma GEMMs [median 28 (95% CI, 27–29) vs. 21 (95% CI, 20–22), Wilcoxon ranked-sum test, *P* < 1.4e−8; Supplementary Fig. S11D], with similar differences by HGSC genotype (Supplementary Fig. S11E) as per gene-level FGA.

Lastly, although expecting that most aneuploidy events observed in our HGSC GEMMs (Supplementary Fig. S13A) are likely passenger alterations due to the genomic instability phenotype, we qualitatively compared the CN profiles observed in our HGSC models with those from seven other studies of HGSC models (*n* = 53) with available genome-wide CN profiles (by array CGH or WGS; refs. 85–91). Importantly, as shown in Supplementary Fig. S13B, these models were generated by a variety of mechanisms (GEMMs, orthotopic cell lines, and *in situ* CRISPR/electroporation), cell of origin (FTE or OSE), and introduced genetics [from large T antigen expression (disrupting *Trp53/Rb1*) to very similar *Trp53/Pten/Brca2*-deficient models]. Despite the wide variation in the amount of aneuploidy (or sub-gene alterations) across models and limited number of tumors profiled per model (*n* = 3–10), the most frequently lost mouse chromosome across the eight other HGSC studies (chr 12) was also the third most frequently lost chromosome in our HGSC models, suggesting that this alteration may be under positive selection across models.

Taken together, these data credential our HGSC GEMM-based tumor models for the relative amounts and patterns of genomic instability found in analogous human tumors; however, additional evaluation would be formally required to evaluate HRD status in these models. In addition, although requiring additional functional evaluation outside of the current study, we observed significant differences in genomic instability within our HGSC models in line with their different latency in HGSC formation, as well as identified recurrent loss of chr 12 across HGSC models.

### MiMouse panel for genomic credentialing of GEMMs: colorectal carcinoma models focusing on aneuploidy

We next used MiMouse to credential the fidelity of two colorectal carcinoma GEMM models using tamoxifen-inducible Cre to inactivate one copy of *Apc* in the presence of activated *Kras* (AK) ± altered *Trp53* (AKP) in epithelial cells of the colorectum (via the *Cdx2* promoter; ref. 45). A total of 38 of 91 tumor samples were these genotypes, passed MiMouse QC metrics, and passed our minimum expected tumor content of 30% [10 FFPE adenomas (five AK and five AKP), 16 FFPE adenocarcinomas GEMMs (14 AKP and two AK), four AKP adenocarcinoma cell line–derived allografts, and eight AKP adenocarcinoma cell lines]. Of the 53 excluded samples, 25 were other genotypes (*Apc*^*flox/flox*^; *Apc*^*flox/+*^*Sox9*^*flox/+*^; *Cdx2*^*flox/flox*^*Braf*^*V600E/+*^) or histology (normal, metastatic, and hyperplastic polyps), and 28 did not pass the tumor content requirement.

Analogous to the initial evaluation of the AKP mice by Tang and colleagues (and earlier studies of models targeting only *APC*) using genotyping and/or IHC (45, 114–116), we initially sought to verify biallelic *Apc* inactivation (through somatic alteration of the second non-floxed *Apc* allele) in AK and AKP tumors with MiMouse using the *Trp53* flox CN as a reference, as these colorectal carcinoma models conditionally lose either both *Trp53* alleles (flox/flox) or only one *Trp53* allele [with the other allele having an oncogenic mutation (flox/R270H)]. By MiMouse, no somatic prioritized *Apc* or *Trp53* mutations were identified in any of the 38 tumor samples, excluding second somatic mutations as the cause of bilallelic inactivation in either gene. Hence, the ratio of *Apc* to *Trp53* flox site log_2_ CNR, assuming biallelic inactivation of *Apc*, is expected to be 1 (1:1) in AKP samples with homozygous *Trp53* deletion (*Apc*^*flox/+*^*Kras*^*LSLG12D/+*^*Trp53*^*ex2-10fl/ex2-10fl*^; AKP^fl/fl^) and 0.5 (1:2) in AKP samples with heterozygous *Trp53* deletion of *Trp53* (*Apc*^*flox/+*^*Kras*^*LSLG12D/+*^*Trp53*^*ex2-10fl/p53R270H*^; AKP^fl^); AK samples (*Apc*^*flox/+*^*Kras*^*LSLG12D/+*^) without *Trp53* deletion served as a negative control with an expected ratio of 0 (*Apc to Trp53* flox site log_2_CNR of 0:1). As shown in Supplementary Fig. S14, by regression analysis, the *Trp53* to *Apc* flox site log_2_ CNR met these expectations across AK, AKP^fl/fl^, and AKP^fl^ mice, supporting biallelic copy loss of both *Apc* (in AK and AKP) and *Trp53* (in AKP) in these colorectal carcinoma tumor models.

Lastly, given the differences in the amount of FGA (Fig. 5A) and aneuploidy (Supplementary Fig. S10) between human HGSC and colorectal carcinoma, we compared the aneuploidy profiles of our mouse HGSC and colorectal carcinoma GEMMs. As shown in Supplementary Fig. S15, HGSC GEMMs have significantly greater number of aneuploidy events per sample than colorectal carcinoma GEMMs [HGSC (*n* = 113) vs. colorectal carcinoma (*n* = 16), median 2 (range, 0–10) vs. 0 (range, 0–2), Wilcoxon rank-sum test *P* = 0.0015]. Although three of 16 (19%) colorectal carcinoma GEMM samples had aneuploidy events compared with zero of 10 colorectal GEMM adenoma samples, this difference was not statistically significant (Fisher exact test *P* = 0.26; Supplementary Fig. S15). Notably, the relative contributions of focal events to FGA in our HGSC and colorectal carcinoma GEMMs were similar to what is observed between human tumors (from TCGA; Supplementary Fig. S16A and S16B).

### MiMouse panel for genomic credentialing: cross-species comparison of aneuploidy events between human and mouse colorectal carcinoma

As described above, although lacking focal high-level amplifications and having low FGA, human colorectal carcinoma shows highly recurrent chr 18 loss (a late event between the adenoma/carcinoma transition) and gains of chrs 20p, 20q, 13q, 7p, 7q, and 8q (Supplementary Fig. S10; refs. 46–48), with the fidelity of these events poorly understood in mouse colorectal carcinoma models. Importantly, comparison of aneuploidy events between human and mouse models must incorporate synteny—the mapping of genomic blocks between species based on orthologous genes and their relative order—to inform on the importance of recurrent aneuploidy events as true driving events and localize key gene(s) (26). For example, although *APC* (chr 5) and putative TSGs on chr 18q (*SMAD2*, *SMAD4*, and *DCC*) are located on different human chrs, these blocks localize to mouse chr 18 and are only ∼42 Mb apart (*Apc* to *Smad4*; Fig. 5B). Therefore, we first characterized recurrent aneuploidy events in our mouse colorectal carcinoma models using the entirety of the colorectal carcinoma GEMM and cell line adenocarcinoma cohort (Fig. 5C). As expected, colorectal carcinoma cell line models had increased focal and aneuploidy FGA contributions compared with GEMMs [and comparable with HGSC GEMMs (Supplementary Fig. S16A and S16B); refs. 117, 118]; however, colorectal carcinoma cell line and GEMM models were of the same genotype and as expected (119) shared similar aneuploidy events, supporting their inclusion in a synteny-based analysis to identify regions under positive selection in these models.

Across the 28 colorectal adenocarcinoma samples, chr-level aneuploidy events were tested for significance using a permutation test (see “Materials and Methods”). We identified significant chr loss of mouse chr 14 [21% (six of 28); *q* value = 0] and chr 18 [18% (five of 28); *q* value = 0], whereas chr 5 was the only significantly gained chr [46% (13/28); *q* value = 0; Fig. 5C]. Focusing first on CN losses, as described above, although *APC* (chr 5) and the three putative TSGs *SMAD2*, *SMAD4*, and *DCC* (chr18) are located on different human chrs, they are both located on mouse chr 18. Hence, we hypothesized that in addition to aneuploidy-based loss of *Smad2*, *Smad4*, and *Dcc* in these five colorectal carcinoma tumors, as all our colorectal carcinoma tumors had two copy loss of the floxed *Apc* allele, the somatic *APC* copy loss event may also have affected the three known TSGs. However, as MiMouse only targeted *Smad4*, our gene-level analysis was limited to just this gene. In support of our hypothesis, 14 of 38 (37%) of adenomas and adenocarcinomas had a single copy loss at the gene level of *Smad4* (Fig. 5C), with *Smad4* inactivation (by single-gene copy loss, aneuploidy, or prioritized mutation) occurring more often in invasive carcinomas versus adenomas [14 of 28 (50%) vs. one of 10 (10%), OR = 8.5, one-sided Fisher exact *P* = 0.03]. Furthermore, focal *Smad4* single copy loss occurred more often than expected compared with the loss of other oncogenes, TSGs, and cancer-related genes (Z-proportion test: *P* value = 2.2e−5), supporting the fidelity of *Smad4* loss in our colorectal carcinoma mouse models. Taken together, these cross-species results further support *Smad4* as potential target of chr 18 loss in the adenoma to colorectal carcinoma transition and highlight how synteny must be considered in cross-species analyses of aneuploidy, given the differing genomic arrangement of two of the most frequently altered TSGs *(Apc* and *Smad4*) in human colorectal carcinoma.

**Figure 6.**
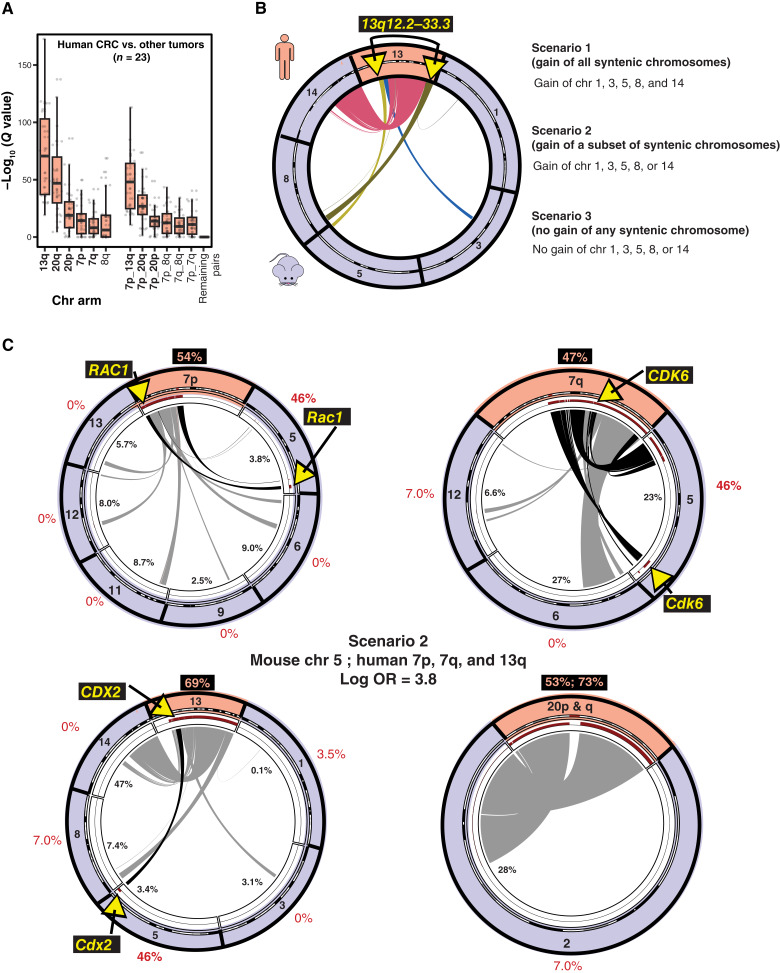
**A,** Cross-species analysis incorporating synteny prioritizes regions for driver gene localization in colorectal carcinoma. To identify colorectal carcinoma specific, we compared arm-level gains (aneuploidy) in human TCGA colorectal carcinoma samples vs. all (*n* = 23) other tumor types. Boxplots of FDR (*Q* value)-corrected statistical significance (negative log *Q* values from Fisher exact test) of arm-level gains (left) and co-occurring aneuploidy events (right) in colorectal carcinoma vs. other tumor types. Those in bold were more frequent in colorectal carcinoma than at least 50% of other tumor types. **B,** Utilization of synteny to prioritize regions of aneuploidy for driver gene identification. Through WGS of human colorectal carcinoma, Cornish and colleagues identified three “peaks” (from 13q12.2-33.3) in the recurrent 13q aneuploidy event. Circos plot showing that due to synteny, this human region (peach) maps to five different mouse (lavender) chrs (1, 3, 5, 8, and 14; syntenic mapping colored separately). Three scenarios could be expected in mouse models of human colorectal carcinoma–specific/enriched arm-level gains (example for human 13q is shown): gain of all syntenic chrs if genes across the arm are functionally significant (scenario 1); gain of only a subset of syntenic chrs (if only one or a limited number of genes are functionally significant; scenario 2); or no gain of any syntenic chrs (if that event is not conserved in the mouse model). **C,** To prioritize potential driver genes from peak regions and the fidelity of human-specific colorectal carcinoma aneuploidy gains (from **A**) to MiMouse-identified aneuploidy gains in our colorectal carcinoma tumors (Fig. 5C), we performed permutation tests to evaluate scenarios 1–3. Circos plots for each human chr (peach) show all syntenic mouse chromosomes (lavender). The percentage of synteny of each mouse chr to the human chr is shown inside the Circos plot. The aneuploidy (gain) frequency of each syntenic mouse chr in colorectal carcinoma samples is shown outside the Circos plot; those significant (from Fig. 5C) are shown in bold with syntenic mapping in black. By permutation testing, only scenario 2 for mouse chr 5 (syntenic chr 7p, 7q, and 13q) was found to be more likely than scenario 1 or 3 (scenarios 1 and 3 were equally likely). Candidate driver oncogenes (from colorectal carcinoma or pan-tumor studies as reported by Cornish and colleagues ; ref. 46), as shown in yellow, were prioritized by intersection of the syntenic human blocks with peaks of CN gains (from these chr) in the WGS study of >2,000 human colorectal carcinoma. Most notably, *CDX2* (human 13q.12.2), which maps to the gained region of mouse chr 5, is both a reported colorectal carcinoma lineage-specific oncogene but is also the source of the promoter (*Cdx2*-P) used to drive transgene expression in our colorectal carcinoma models. CRC, colorectal carcinoma.

We next sought to evaluate the fidelity of human colorectal carcinoma copy gains in our mouse models. As described above, not only are gains of chrs 20p, 20q, 13q, 7p, 7q, and 8q (46–48) common in human colorectal carcinoma, we found that when compared with other solid tumor types in TCGA (*n* = 23), chr arms 13q (Fisher exact: *q* val range: 2e−173 to 4.7e−20; OR range, 4–200) and 20q (Fisher exact: *q* val range: 2e−140 to 8.1e−7; OR range, 2–515) gains were significantly more frequent in colorectal carcinoma than any of the 23 other cancer types (Fig. 6A). Chr arms 20p, 7p, and 7q gains were also more frequent in 87% (20 of 23), 65% (15 of 23), and 65% (15 of 23) of other cancer types. Of interest, when considering pairwise combinations, only the combination of 7p with either 13q or 20q was observed more often in colorectal carcinoma compared with all other solid tumors (Fig. 6A). Highlighting the potential impact of synteny on localizing driver genes to these regions, at least three separate “peaks” have been reported in an ∼80 Mb region (from 13q12.2 to 13q33.3; ref. 46) in the colorectal carcinoma–specific 13q gain, with this region (and candidate driver genes; refs. 46–48, 120) mapping to mouse chr 14 (containing *KLF5* and *CUL4A*), chr 8, chr 5 (containing *CDX2* and *CDK8*), chr 3, and chr 1 (Fig. 6B).

Therefore, incorporating synteny, we modeled how well the observed mouse arm levels gains in our colorectal carcinoma models (Fig. 5C) matched the expected results based on the human colorectal carcinoma gains of chrs 7p, 7q, 13q, 20p, and 20q using the following scenarios: (i) gain of all syntenic mouse chrs (if genes across the length of the human chr are functionally important), (ii) gain of only a subset of syntenic mouse chrs (if only one or a limited number of genes on the human chr are functionally important/dominant), or (iii) no gains in syntenic mouse chrs (Fig. 6B). We tested all three scenarios for the human chrs of interest (Fig. 6C), using a permutation test (logOR as a test statistic; scenario 1 as the reference compared with scenarios 2 and 3). In all human chr arms of interest, there was no significant difference in the occurrence of no gains of any of the syntenic chrs (scenario 3) compared with gain of all syntenic chrs (scenario 1; *P* value range = 0.74–1.0). Gains of a subset of syntenic chrs (scenario 2) were found to be more likely to occur than gain of all syntenic chrs (scenario 1), but only for gain of mouse chr 5, which is syntenic to human 7p, 7q, and 13q, with a median log OR of 3.8 (*P* values = 0).

Of note, all three human chr arms syntenic to mouse chr 5 are concurrently gained in 37% (221 of 616) of TCGA colorectal carcinoma samples (Supplementary Fig. S10), and concurrent 7p and 13q gain were found to co-occur more frequently in colorectal carcinoma than any other solid tumor type (Fig. 6A). As these results suggest that the functional drivers of the human aneuploidy are most likely to be located in the syntenic regions mapping to mouse chr 5, we evaluated these syntenic regions versus “peaks” (by GISTIC2.0) of CN gains from the Cornish and colleagues WGS study of >2,000 human colorectal carcinoma [Supplementary Table S16 from (46)]. Interestingly, peaks from 7p22.3 (chr 7:1–876,040, in microsatellite stable metastases) and 13q12.2 (chr 13:27,909,137–27,999,097 and chr 13:27,024,690–28,182,919 in microsatellite-stable primary and metastases) intersect the regions syntenic to mouse chr 5 (Fig. 6). Although ∼12% and 8% of human 7p and 13q, respectively, map to mouse chr 5, these peak regions constitute only 4% (7p22.3) and 1% (13q12.2) of their total arm lengths, suggesting that these arm-level events may function to alter the expression of a limited set of genes contained in this region of recurrent gain in our mouse model.

To explore the potential impact of these genes, we looked at comprehensive gene knockout studies via CRISPR using DepMap (https://depmap.org/portal; ref. 99). The syntenic block 13q12.2 contains 39 genes, with only seven genes having at least one colorectal carcinoma cell line showing relevant gene dependency scores (≤ −1; Supplementary Fig. S17A). Interestingly, when comparing the dependency scores of these seven genes in colorectal carcinoma cell lines with those of 17 other cancer cell lines, only *CDX2* was more significantly essential in colorectal carcinoma (*q* value <0.05), consistent with previous reports that *CDX2* acts as a lineage-specific oncogene in the context of colorectal carcinoma cell lines (Supplementary Fig. S17B; ref. 120). Although on average the dependency scores for *CDX2* were lower than other cancer types, only 13% (7/53) of the colorectal carcinoma cell lines had a relevant dependency score for *CDX2*. These seven cell lines had significantly higher expression of *CDX2* than the other 46 colorectal carcinoma (Wilcoxon rank-sum test *P* = 0.01), with four of seven also having chr 13q gain (the remaining three samples did not have CN status data). Among the colorectal carcinoma cell lines, there was no significant difference in the gene dependency score based solely on 13q being gained (Wilcoxon rank-sum test *P* = 0.1; Supplementary Fig. S17C). Of note, *Cdx2* is also the promoter used to drive transgene expression in our conditional AKP models. These results, like those with *SMAD4* and *APC*, highlight another layer of complexity that must be considered in the generation and analysis of mouse models of human cancer due to synteny. Taken together, through profiling with a first-generation mouse CGP panel and incorporation of synteny, our results support AK and AKP as high-fidelity models of the neoplastic progression of human colorectal carcinoma through biallelic *Apc* and *Trp53* loss, shared loss of S*mad4* during the transition from adenoma to adenocarcinoma, and conserved (syntenic) aneuploidy-based CN profiles.

## Discussion

To address the need for comprehensive genomic credentialing of mouse tumor models (particularly GEMMs), herein, we describe considerations specific to mouse CGP development, designed a 0.5 Mb mouse CGP panel (MiMouse) applicable to FFPE tissues, and profiled more than 200 mouse tumors from colorectal carcinoma and HGSC cancer models using MiMouse. Although nearly all human tumor types have been comprehensively genomically characterized (many with WGS of 100–1,000s of tumors; refs. 24, 46–48) and clinical CGP testing from FFPE tissue is widespread (13, 33), the majority of mouse tumor models have been poorly characterized by comprehensive genomic approaches. Importantly, although fresh frozen tumor tissue is more routinely available for mouse tumor models than for human patients, mouse-specific approaches applicable to FFPE tissue enable more precise isolation of tumor (vs. non-neoplastic elements), as well as characterization of the full range of neoplasia (from precursor lesion to metastases), for which mouse models are uniquely suited despite the minute lesion size (and isolatable genomic DNA). Given that most mouse models force simultaneous expression of a limited set of alterations using a single promoter, genomic credentialing through approaches such as MiMouse may enable more precise model selection as part of more general precision medicine approaches.

To adapt human approaches to a mouse CGP panel, several cross-species issues require careful consideration, including cross-species mutation mapping (to identify driving alterations), accounting for mouse strain–specific variations that affect observed phenotypes, and dealing with the frequent inbreeding and/or backcrossing in mouse models. For instance, GEMMs designed to model intestinal carcinomas driven by mismatch repair deficiency (through knockout of both *Msh6* alleles) have varying phenotypes depending on the strain background: mice with a mixed background of C57BL/6J and 129S1/SvmJ produced both adenomas and carcinomas, whereas a congenic C57BL/6 mouse only produce adenomas, and the mixed background of 129/OLA and FVB/NJ rarely produced any neoplasia (121). Likewise, commonly used inbred mice show extensive strain-specific haplotype variation, with the most diverse regions enriched for immune- and pathogen defense-related genes (122).

Herein, we provide a simple approach for evaluating the functional significance of mouse model somatic mutations and demonstrate through simulation that sets of 500 to 1,000 SNPs can effectively determine basic strain background from tumor-only sequencing (although comprehensive assessment from nontumor tissue is routine by genotyping arrays). Although MiMouse showed high performance on strain detection (when >5% present) in our HGSC and colorectal carcinoma model systems, future approaches could improve performance by incorporating mouse reference haplotypes and excluding SNPs in regions of homopolymers [a known limitation of Ion Torrent sequencing (39) that resulted in a loss of MiMouse genotyping SNPs] or incorporating background error correction approaches and/or alternative sequencing platforms.

Our results herein highlight the trade-offs between breadth, cost, and applicability to minute amounts of input DNA that must be considered when considering the use of tumor-only CGP (as well as the composition of panels such as MiMouse) versus WES/WGS for mouse model credentialing, analogous to those in human translational and clinical applications. We designed the MiMouse panel to have broad applicability for initial genomic credentialing, although specific choices were informed by the colorectal carcinoma (microsatellite stable) and HGSC models available to us. For example, given the rarity of high TMB in these human tumors, our panel does not have a sufficient genomic footprint to reliably estimate TMB around the human clinically relevant threshold of 10 Muts/mb (103). Furthermore, although HGSC is characterized by whole-genome doubling and significant genomic instability, the composition of the MiMouse panel (and lack of uniformly spaced amplicons across the genome) limited its ability to accurately call sub-chromosomal CNAs that are particularly indicative of HRD (52, 54, 55, 108). However, our primary interest was in evaluating general genomic instability (for which we demonstrated that our panel was adequate through multiple methods of evaluating FGA) in the HGSC GEMMs, and whole chr gain/loss in colorectal carcinoma models (in which the most recurrent gains/losses in humans are arm level; refs. 17, 46–48).

Importantly, subsequent versions of MiMouse can be easily expanded based on the simulations performed herein to increase strain resolution (using additional NGS-platform optimized and haplotype discriminating SNPs) and resolution of sub-chromosomal CNAs (using more amplicons for both single genes and marker density; ref. 108), as well as incorporating additional desired genes of interest for mutations/CNAs and precise evaluation of TMB (by expanding the genomic footprint to at least 0.8 Mb; ref. 103). In anticipation of an expanded gene set, and to provide a reference for those generating mouse CGP panels, we have determined all prioritized conserved residues (in which nonsynonymous mutations would be prioritized by our conservation score) across the expanded set of OncoKB genes (Supplementary Table S7). Furthermore, an expanded panel would enable purity and ploidy estimations, as with other CGPs and their companion algorithms (like MSK-IMPACT/FACETS) which can be used with a reduced number of SNPs from a 1.5 Mb panel [15,000 SNPs (after QC) and a floor of 10% heterozygosity rate; ref. 123]. Hence, we envision MiMouse (or other similar CGP panels) as most applicable for rapid, inexpensive credentialing of both precursor and localized/metastatic tumors that can be used to guide more comprehensive evaluation in initially promising models. Our identification of rare somatic mutations/amplifications in established oncogenes in both HGSC and colorectal carcinoma models highlights the potential need to screen many individual tumors to identify additional somatic driver gene alterations in GEMMs.

The application of MiMouse to an HGSC model system [using tamoxifen-inducible Cre to inactivate *Trp53* with various combinations of *Brca1*, *Rb1*, and *Nf1* in epithelial cells of the murine oviduct (via the Ovgp1 promoter); ref. 56] largely confirmed the results of our previous study, which employed a much smaller, HGSC-focused panel (31). Consistent with our earlier findings, oncogenic mutations and high-level CNAs were exceptionally rare, while high genomic instability (by multiple measurements of FGA) was present, mirroring the dominant genomic characteristic of human HGSC. Importantly, the expanded MiMouse panel reduced the overestimation of percent genome altered that we observed with our smaller panel, thereby providing a more accurate measure of genomic instability/FGA in these models. Given the role of genomic instability in HGSC development and therapeutic response, accurate genomic credentialing of preclinical models is essential to ensure fidelity to this defining genomic characteristic. Lastly, and although requiring additional functional evaluation beyond the scope of this study, we observed significantly less genomic instability in the HGSC model with the shortest latency, suggesting that such instability may accumulate consistently during tumorigenesis in these models, as well as confirmed recurrent loss of mouse chr 12 across HGSC models both from our study and previously published HGSC models varying in introduced genetics, methodology, and oviductal cell of origin.

In the colorectal carcinoma model system [tamoxifen-inducible Cre to activate *Kras* with inactivation of *Apc* (AK) ± altered *Trp53* (*AKP*) in epithelial cells of the colorectum (via the *Cdx2* promoter); ref. 45] evaluated by MiMouse, oncogenic mutations and high-level CNAs were also exceptionally rare, although we identified prioritized *Smad4* and *Pik3ca* mutations and *Pik3ca* and *Myc* amplifications, with these being among the most frequent mutations/amplifications in human microsatellite-stable colorectal carcinoma (beyond those genetically altered in our mouse models; refs. 46–48). More importantly, through our assessment of arm-level CNAs, we made additional insights into shared and species-specific recurrent aneuploidy (single copy chr level gain or loss) events in human colorectal carcinoma and mouse models, which are particularly relevant as human microsatellite-stable colorectal carcinoma shows an aneuploidy-dominated structural variant signature (11). Notably, this cross-species syntenic analysis enabled evaluation of fidelity at both the chr level, as well as in prioritization/identification of regions harboring potential driver genes (if the human aneuploidy event maps to multiple mouse chrs or *vice versa*). With regard to recurrent losses, we identified copy loss of the second (non-floxed) *Apc* allele in all samples (from adenoma to metastatic carcinoma), consistent with genotyping results from earlier studies and the essentiality of biallelic *Apc* loss for neoplastic initiation (114–116). Additionally, although the majority of human chr 18 (∼80%) maps to mouse chr 18, aneuploidy-based loss of mouse chr 18 was only present in a subset of cell line models (five of 28); however, although *Apc* is located on human chr 5, in mice, it is located on chr18, only separated by 42 Mb from the 5 Mb block genomic region containing known/putative TSGs (*Smad2*, *Smad4*, and *Dcc*) and could potentially be affected by the same copy loss event. Although MiMouse did not profile *Smad2* or *Dcc*, in support of our hypothesis, 50% (14 of 28) of colorectal adenocarcinoma samples had single-copy *Smad4* loss [vs. one of 10 (10%) of adenomas], supporting the fidelity of the AKP model to *Smad4* single-copy loss, although through a different mechanism than in human tumors due to synteny. Together, these results credential this model as reflective of the importance of *SMAD4* in the adenoma to carcinoma transition and support additional experimentation in future studies to determine whether a single event is responsible for simultaneous *Apc* and *Smad4* loss in AKP mice.

Likewise, we only identified a singular recurrent chr gain in our colorectal carcinoma models, with chr 5 gain occurring in zero of 10 and 13 of 28 of adenomas and carcinomas, respectively. Overall, our adenoma samples lacked any instance of aneuploidy, which is consistent with the increased frequency of CN events in human carcinoma versus adenoma (124). Notably, human colorectal carcinoma is characterized by recurrent chromosomal gains (e.g., 20p, 20q, 13q, 7p, 7q, and 8q; refs. 46–48), and we show the former three arm-level gains occur more frequently in colorectal carcinoma than any other solid tumor. Although we found two events syntenic to chr 20p or 20q gain (gain of mouse chromosome 2), our permutation test did not find the gain of mouse 2 to be significant. The lack of significance could stem from the small cohort size. The rest of our synteny-based analysis identified significant support for gains in human 7p, 7q, and 13q, which all map to mouse chr 5. Among the syntenic blocks between these regions, *CDX2*, *CDK8*, *GSX1*, and *PDX1* are among candidate colorectal carcinoma and/or cancer driver genes affected by these recurrently gained chrs between human and mouse. Among these genes, we observe only *CDX2* to be a colorectal carcinoma–specific essential gene by DepMap (99). Of note, in the absence of clear driver genes also covered by MiMouse, we cannot definitively evaluate the scenario of sub-chromosomal gains in syntenic regions or single genes; however, outside of *Myc*, which had a low-level amplifications in eight of 28 colorectal carcinoma GEMM models (which is a candidate driver from the human 8q recurrent aneuploidy event), the lack of such events in human colorectal carcinoma and the overall fidelity of aneuploidy-based CN profile in the GEMM model make this scenario less important. Similarly, although *CDX2* is a functionally supported candidate driver (for specific cell lines) in the broad human chr 13 aneuploidy event (120) that maps to the recurrent chr 5 gain in our mouse models, the *Cdx2* promoter is also used to drive all genetic events in the AKP model system. Such considerations, in addition to the engineered genetic alterations in GEMMs, highlight the importance of functional studies in validating aneuploidy drivers (using systems such as ReDACT; ref. 27), as well as the potential utility of parallel evaluations of both human and mouse systems in which synteny can be leveraged to isolate dosage of candidates on different mouse chrs. For example, in a high-fidelity mouse model representative of human 1q aneuploidy, in which Girish and colleagues (27) found that *MDM4* (1q32.1) and *BCL9* (1q21.2) were both drivers, given that these genes map to different mouse chrs (chr 1 and 3, respectively), we would expect mouse models to gain both chrs (although other mechanisms to increase expression of one or both genes could compensate for lack of gain). Lastly, although we were able to identify recurrent CNA events in our mouse colorectal carcinoma model, roughly a third (nine of 28) of the tumors (despite having ≥30% tumor content) had no detectable aneuploidy or prioritized mutations/focal CN events or *Smad4* loss. Whether this population has no additional somatic driver alterations (beyond those engineered), focal events involving other non-targeted genes, is driven by multiple single-copy level focal gain/losses, or these tumors have more complex genomic alterations will require more high-resolution approaches beyond the scope of the current study.

Herein, we address important cross-species considerations for genomic credentialing of mouse models using an FFPE CGP panel and apply the MiMouse panel (0.5 Mb) to both HGSC and colorectal carcinoma mouse models, providing valuable insights into their genomic fidelity relative to human cancers. Our findings highlight both the strengths and limitations of using targeted CGP panels for credentialing FFPE mouse tumor models and underscore the importance of considering species-specific genomic architecture in translational cancer research. Our results can be used to guide future panel iterations, including expansion of the MiMouse panel to facilitate assessment of clinically relevant TMB cutoffs and finer resolution of CNAs and strain admixture, and highlight the utility of complementary approaches (e.g., low-pass WGS for CN profiling or MGA from non-tumor genomic DNA). More broadly, our results highlight the utility of CGP like approaches—whether by MiMouse or other technologies—to broadly credential the full spectrum of neoplasia (from precursor through metastasis) murine models across potentially relevant characteristics, including strain, driver mutations CNA patterns, and more complex CN/mutation patterns. Similarly, the variability (and rarity) of somatic alterations across tumors within a given model system also argues for profiling a broad spectrum of tumors, including those used to draw major conclusions about the clinical utility of a given model. For example, the association observed in our HGSC models between latency and genomic instability would support profiling all tumors (at sacrifice) from an experiment evaluating the impact of an intervention (vs. control) on tumor development in a given HGSC model to account for the potential impact of genomic instability in individual tumors in both arms. In summary, our results demonstrate the complexity of cross-species comparisons, further highlighting the need for broad genomic credentialing of mouse models to ensure the highest fidelity to human neoplasia (or to provide counterfactual model systems) given their critical role in preclinical research and the increasing focus on the poor translatability of animal models, as exemplified by the NIH no longer seeking research proposals that exclusively involve animal models (125).

## Supplementary Material

Figure S1 Approach for evaluating the functional significance of mutations detected by mouse CGP

Figure S2 Aneuploidy detection considerations for reduced coverage approaches like MiMouse

Figure S3 Aneuploidy detection performance across TCGA tumor types using different Rc thresholds by breadth of chromosome arm coverage ( c )

Figure S4 Coverage characteristics of the MiMouse panel

Figure S5 Aneuploidy detection performance of MiMouse with and without the directionality filter and by variable Rc thresholds and c

Figure S6 Gene level correlation of copy number by MiMouse and MGA

Figure S7 Indels limit informative SNPs from Ion Torrent based MiMouse profiling

Figure S8 Highly correlated detection of focal, sub‐gene deep (homozygous) deletions by MiMouse

Figure S9 MiMouse for CGP and genomic credentialling across the neoplastic spectrum of two large cohorts of inducible fallopian tube (Ovary) and colorectal (CRC) tumors

Figure S10 Aneuploidy events in human HGSC and CRC

Figure S11 FGA differs in human and mouse models of HGSC and CRC

Figure S12 Focused NGS panels overestimate FGA vs. CGP and WES in human HGSC

Figure S13 Comparison of aneuploidy between our HGSC models and other models

Figure S14 CRC tumors from AK and AKP mice show somatic loss of the second Apc allele

Figure S15 Aneuploidy comparison between HGSC and CRC models

Figure S16 Contribution of aneuploidy vs. focal events to FGA in human and mouse CRC and HGSC

Figure S17 Comparison of dependency scores and RNA expression for syntenic region 13q12.2

Table S1 MiMouse panel composition & summary table of markers

Table S2 Engineered mutation detection by MiMouse in colorectal cancer (CRC) samples

Table S3 Table of sequencing statistics, sample information, and strain information for colorectal (CRC) and high grade serous cancer (HGSC) samples

Table S4 Somatic mutations identified in MiMouse profiled colorectal cancer (CRC) and fallopian tube (ovarian) high grade serous cancer (HGSC) and endometrioid carcinoma (EC) tumor samples

Table S5 Clinically relevant mutations and copy number alterations for HGSC and CRC cohorts

Table S6 Gene level copy number calls (log2 copy number ratio [CNR]) by MiMouse for colorectal cancer (CRC) and high grade serous cancer (HGSC) tumor samples

Table S7 Human prioriztized amino acid positions (oncokb) and mouse analog

## Data Availability

The MiMouse profiling data have been submitted to the Short Read Archive and are available as project PRJNA1311633. All code generated from this study is available at https://github.com/khuhu/MiMousePaper. Other data generated in this study are available upon request from the corresponding author.
