# Supplementary material for: Development and Application of MiMouse, a Comprehensive Genomic Profiling Panel for Credentialing Mouse Tumor Models
Source: Cancer Res Commun. 2025 Oct 29;5(10):1910–33. doi: 10.1158/2767-9764.CRC-25-0279 (PMC12569591; doi:10.1158/2767-9764.CRC-25-0279)
Supplement: Figure S1 — Approach for evaluating the functional significance of mutations detected by mouse CGP [file crc-25-0279_figure_s1_suppsf1.pdf]

Figure S1

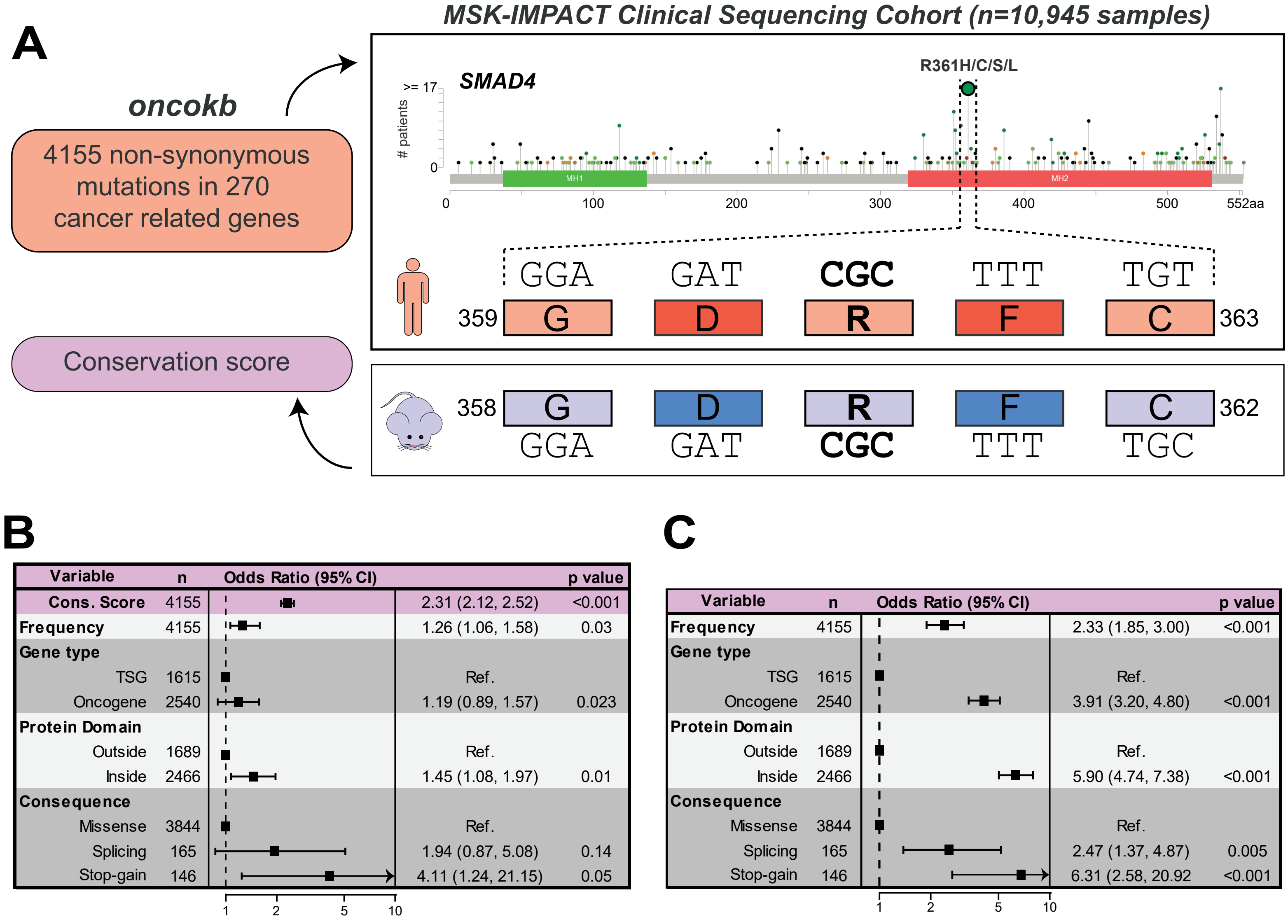

Figure S1. Approach for evaluating the functional significance of mutations detected by mouse CGP. A) Overview of the workflow to determine whether amino acids affected by recurrent mutations (nonsynonymous substitutions of amino acid) in human tumors are conserved in mice. From MSK-IMPACT (n=10,945 samples) pan-tumor human sequencing (6), we converted impacted amino acids from 4,155 nonsynonymous mutations (from 270 cancer genes) to those in mice. Data from *SMAD4* (focusing on amino acid p.R361) is shown, with the lollipop plot from cbioportal showing all somatic mutations. We used two filtering steps to check if the amino acids positions of interest were conserved: (1) the amino acid found in humans was the same as the amino acid at the converted position, (2) the local conservation score (>0) to filter out spurious amino acid matches. **B)** Forest plots depict the odds ratio, confidence intervals and p-values for the effect different factors have on whether a mutational position (amino acid) was conserved between human and mouse when including **(B)** and excluding **(C)** the local conservation score of the alignment. Discrete variables such as gene type, protein domain and mutation consequence were made comparable to continuous variables such as conservation score and frequency by scaling the variables by two times the standard deviation.
