# Supplementary material for: Development and Application of MiMouse, a Comprehensive Genomic Profiling Panel for Credentialing Mouse Tumor Models
Source: Cancer Res Commun. 2025 Oct 29;5(10):1910–33. doi: 10.1158/2767-9764.CRC-25-0279 (PMC12569591; doi:10.1158/2767-9764.CRC-25-0279)
Supplement: Figure S2 — Aneuploidy detection considerations for reduced coverage approaches like MiMouse [file crc-25-0279_figure_s2_suppsf2.pdf]

Figure S2

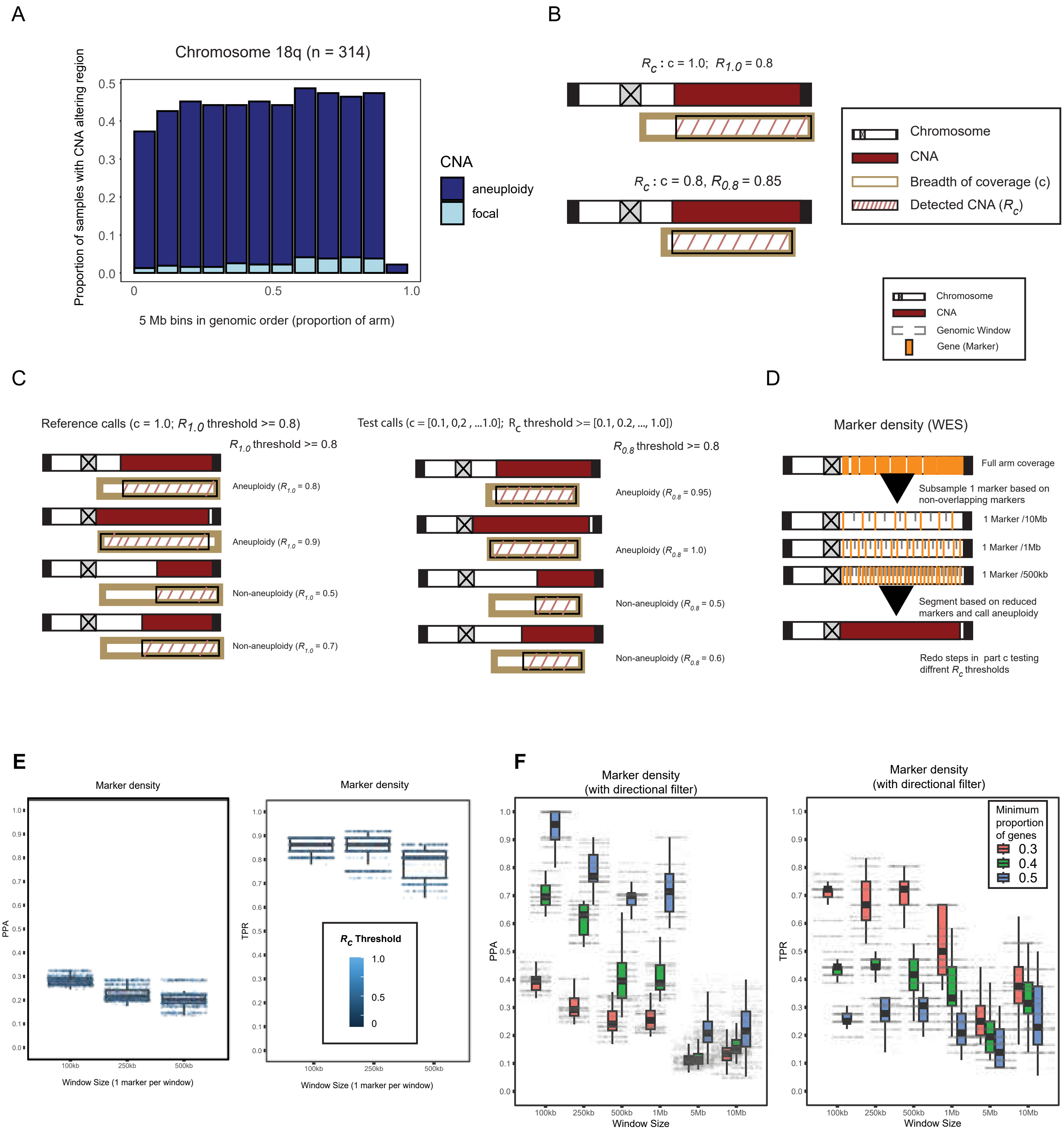

A
Chromosome 18q (n = 314)
Proportion of samples with CNA altering region
5 Mb bins in genomic order (proportion of arm)
CNA
aneuploidy
focal
B
R_c : c = 1.0; $R_{1.0}$ = 0.8
R_c : c = 0.8, $R_{0.8}$ = 0.85
Chromosome
CNA
Breadth of coverage (c)
Detected CNA (R_c)
Chromosome
CNA
Genomic Window
Gene (Marker)
C
Reference calls (c = 1.0; $R_{1.0}$ threshold >= 0.8)
$R_{1.0}$ threshold >= 0.8
Aneuploidy ($R_{1.0}$ = 0.8)
Aneuploidy ($R_{1.0}$ = 0.9)
Non-aneuploidy ($R_{1.0}$ = 0.5)
Non-aneuploidy ($R_{1.0}$ = 0.7)
Test calls (c = [0.1, 0,2 , ...1.0]; R_c threshold >= [0.1, 0.2, ..., 1.0])
$R_{0.8}$ threshold >= 0.8
Aneuploidy ($R_{0.8}$ = 0.95)
Aneuploidy ($R_{0.8}$ = 1.0)
Non-aneuploidy ($R_{0.8}$ = 0.5)
Non-aneuploidy ($R_{0.8}$ = 0.6)
D
Marker density (WES)
Full arm coverage
Subsample 1 marker based on non-overlapping markers
1 Marker /10Mb
1 Marker /1Mb
1 Marker /500kb
Segment based on reduced markers and call aneuploidy
Redo steps in part c testing diffrent R_c thresholds
E
Marker density
PPA
Window Size (1 marker per window)
Marker density
TPR
R_c Threshold
Window Size (1 marker per window)
F
Marker density (with directional filter)
PPA
Window Size
Marker density (with directional filter)
TPR
Minimum proportion of genes
Window Size

Figure S2. Aneuploidy detection considerations for reduced coverage approaches like MiMouse.
A) To address the fundamental limitation of aneuploidy detection by a targeted NGS approach (the inability to detect the true size of CNAs from reduced breadth of coverage [$_c$]), we first leveraged the observation by Taylor *et al.*, who found that in pan-tumor human TCGA data, arm-level events generally altered at least 80% of the chr arm (in the same direction)(2). We hypothesized that the ability of MiMouse (and other reduced coverage [$_c$ <1] approaches) to detect arm-level alterations could be evaluated by determining the proportion of a chr arm being altered from its midpoint. The histogram shows the proportion of human TCGA CRC samples (diploid) from TCGA, where an aneuploidy (dark blue) or interstitial CNA (light blue) alters the entirety of a 5Mb genomic window in human chr 18q. The 5Mb windows are sorted in genomic order. **B)** To simulate the effects of reduced coverage by CGP vs. near genome-wide technologies, we developed a metric, R_c , where $_c$ is the breadth of coverage for a chr arm (gold) anchored at the midpoint of the chr arm, and R_c is the proportion of the chr arm altered (hatched maroon region) by a copy number alteration (CNA; maroon) specific to $_c$. The resulting R_C for the same CNA measured by assays with different $_c$ ($R_{1.0}$ = near-genome wide) is shown. Centromeres are indicated by an X. **C)** Diagram showing our simulations using different R_c thresholds to call aneuploidy in diploid TCGA tumors (n = 6,591) while also varying $_c$. The reference set was made with $R_{1.0}$ >= 0.8 based on the findings of Taylor *et al*(2). **D)** Diagram showing simulation of marker density using CRC cell lines (n = 9) from CCLE sequenced with WES. Non-overlapping genomic windows of varying sizes (grey) i.e 100kb, 250kb were made for each chromosome and a gene or marker (orange), with genes preferred, was randomly chosen for each window. Aneuploidy was called using the method described in **C)** with reference calls being made with all targeted markers. **E)** Boxplots of the positive percent agreement (PPA) and true positive rate (TPR) for aneuploidy calls made in **D)** for the various genomic windows without any additional filtering. Different R_c thresholds were also tested to see if any would improve the calls (indicated by the gradient of blue). **F)** Boxplots of PPA and TPR for results in **E)** with our directional filter added, and colored by the minimum proportion of genes required to be altered in the same direction of the called aneuploidy (gain or loss) by the filter.
