# Supplementary material for: Development and Application of MiMouse, a Comprehensive Genomic Profiling Panel for Credentialing Mouse Tumor Models
Source: Cancer Res Commun. 2025 Oct 29;5(10):1910–33. doi: 10.1158/2767-9764.CRC-25-0279 (PMC12569591; doi:10.1158/2767-9764.CRC-25-0279)
Supplement: Figure S3 — Aneuploidy detection performance across TCGA tumor types using different Rc thresholds by breadth of chromosome arm coverage ( c ) [file crc-25-0279_figure_s3_suppsf3.pdf]

# Figure S3

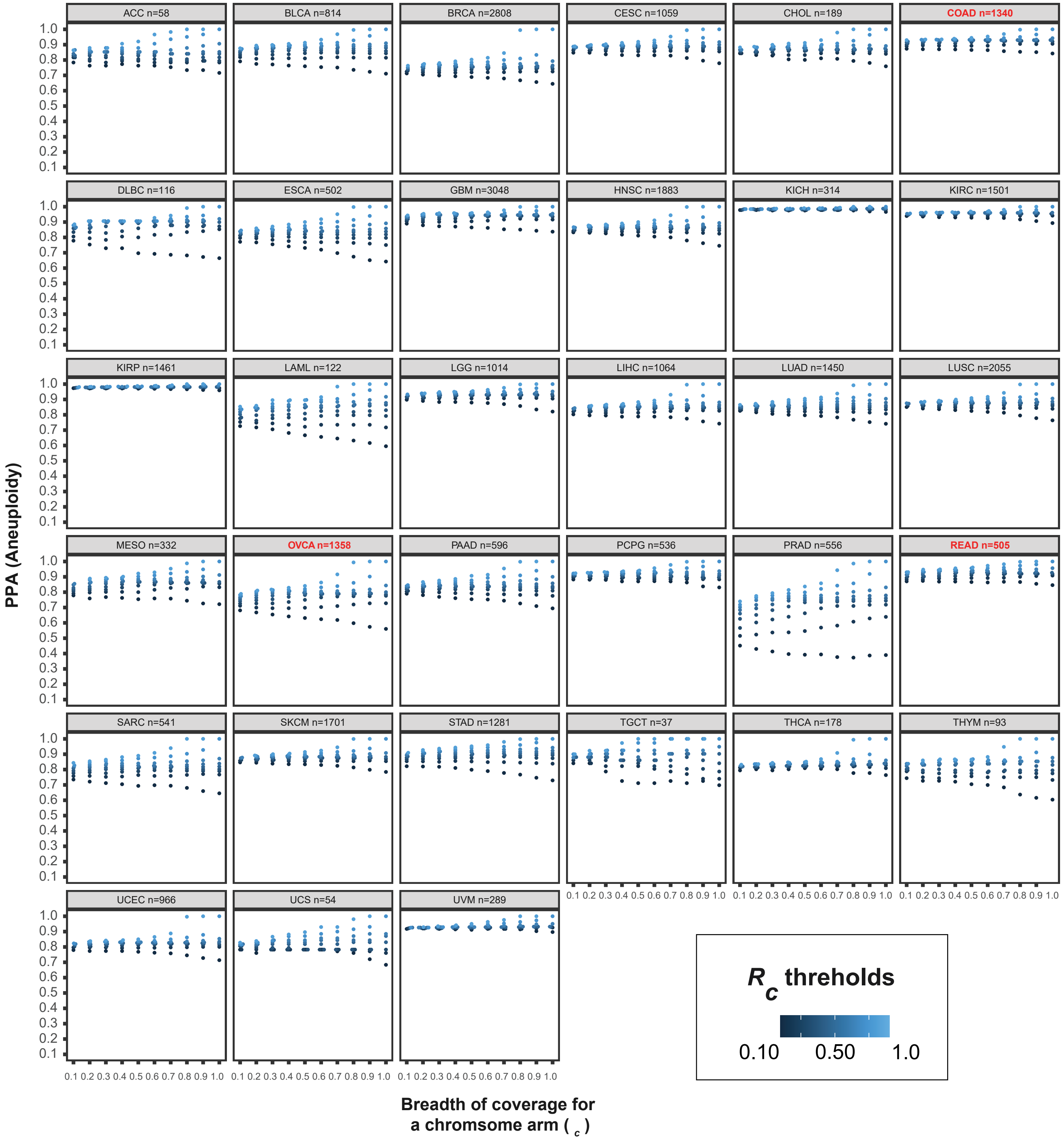


**Figure S3. Aneuploidy detection performance across TCGA tumor types using different $R_c$ thresholds by breadth of chromosome arm coverage ( $_c$ ).**
Dotplots of positive percent agreement (PPA) for simulations as described in **Fig S2C** and shown in **Figure 2B**, except stratified by cancer type. HGSC (OVCA) and CRC (COAD + READ) human tumors are shown in red.
