# Supplementary material for: Development and Application of MiMouse, a Comprehensive Genomic Profiling Panel for Credentialing Mouse Tumor Models
Source: Cancer Res Commun. 2025 Oct 29;5(10):1910–33. doi: 10.1158/2767-9764.CRC-25-0279 (PMC12569591; doi:10.1158/2767-9764.CRC-25-0279)
Supplement: Figure S4 — Coverage characteristics of the MiMouse panel [file crc-25-0279_figure_s4_suppsf4.pdf]

Figure S4

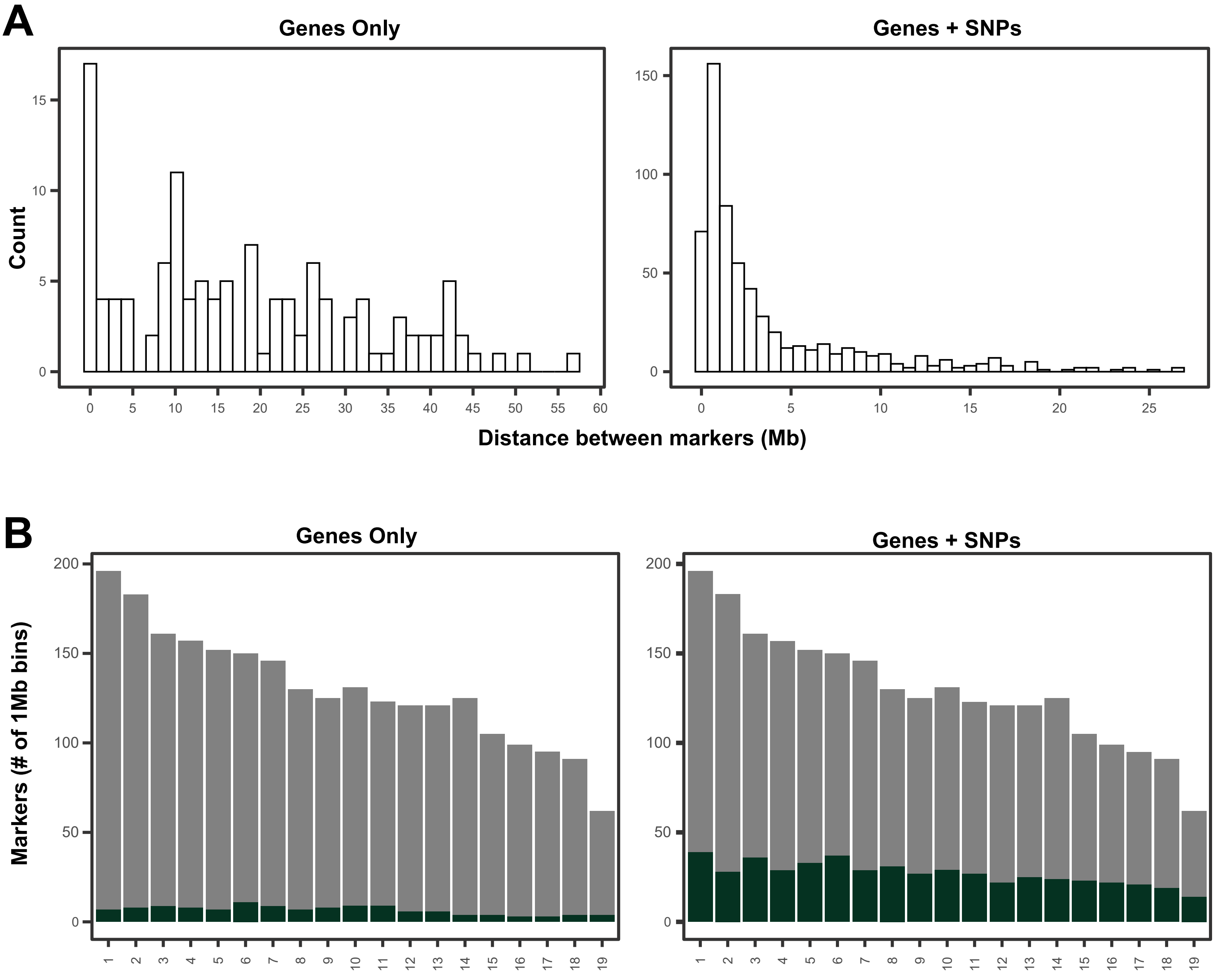

Figure S4. Coverage characteristics of the MiMouse panel.
A) Histograms show the average distance between markers in MiMouse when using genes only (left) and both genes and genotyping SNPs (right). **B)** Stacked histograms showing the number of non-overlapping 1 Mb bins per mouse chromosome with (green) and without (grey) markers based on MiMouse coverage of only genes (left), and inclusion of both genes and SNPs (right).
