# Supplementary figures and images for: Development and Application of MiMouse, a Comprehensive Genomic Profiling Panel for Credentialing Mouse Tumor Models
Source: Cancer Res Commun. 2025 Oct 29;5(10):1910–33. doi: 10.1158/2767-9764.CRC-25-0279 (PMC12569591; doi:10.1158/2767-9764.CRC-25-0279)

# Figure S5

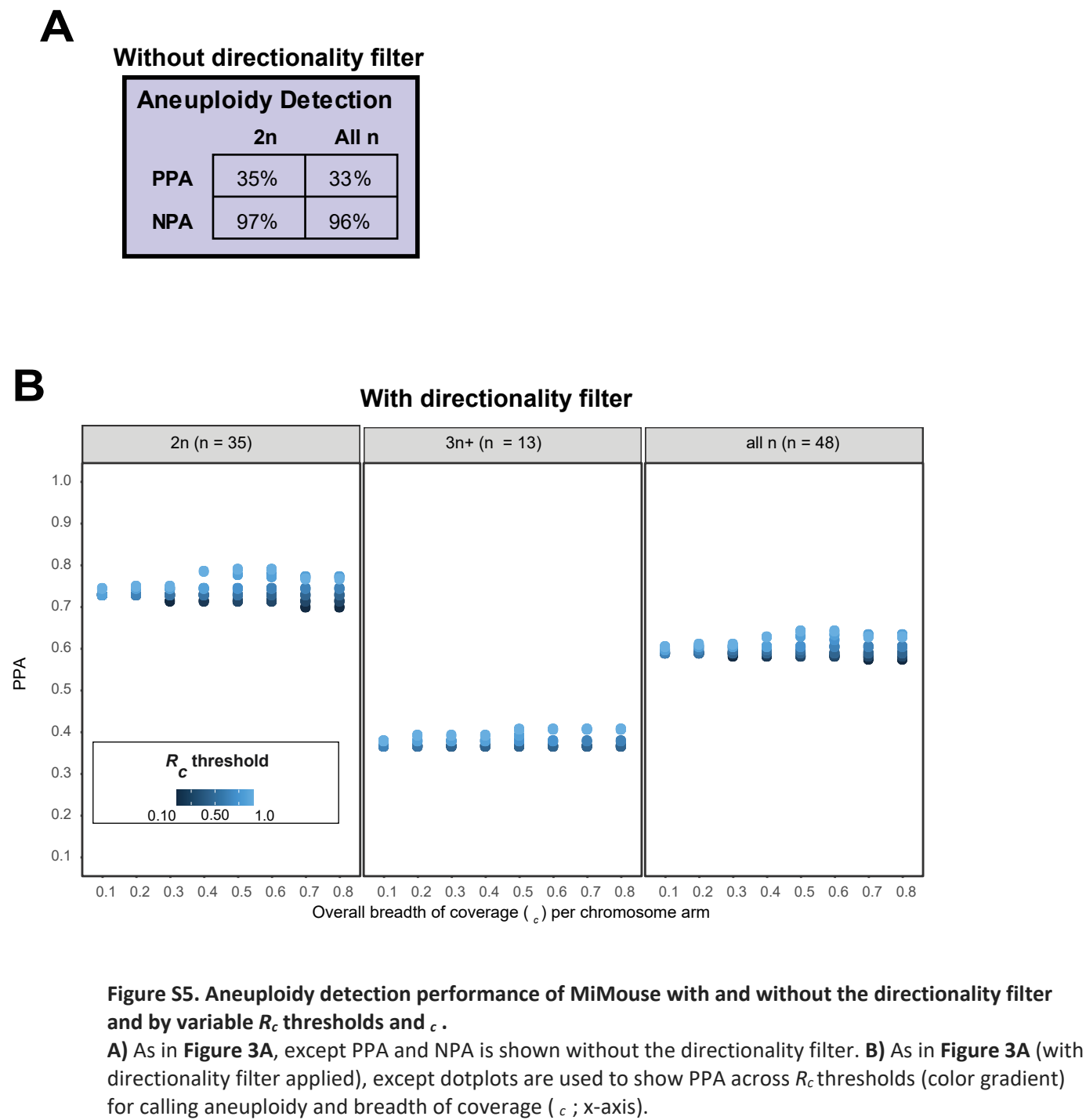

Supplement: Figure S5 — Aneuploidy detection performance of MiMouse with and without the directionality filter and by variable Rc thresholds and c [file crc-25-0279_figure_s5_suppsf5.pdf]
