# Supplementary material for: Development and Application of MiMouse, a Comprehensive Genomic Profiling Panel for Credentialing Mouse Tumor Models
Source: Cancer Res Commun. 2025 Oct 29;5(10):1910–33. doi: 10.1158/2767-9764.CRC-25-0279 (PMC12569591; doi:10.1158/2767-9764.CRC-25-0279)
Supplement: Figure S6 — Gene level correlation of copy number by MiMouse and MGA [file crc-25-0279_figure_s6_suppsf6.pdf]

Figure S6

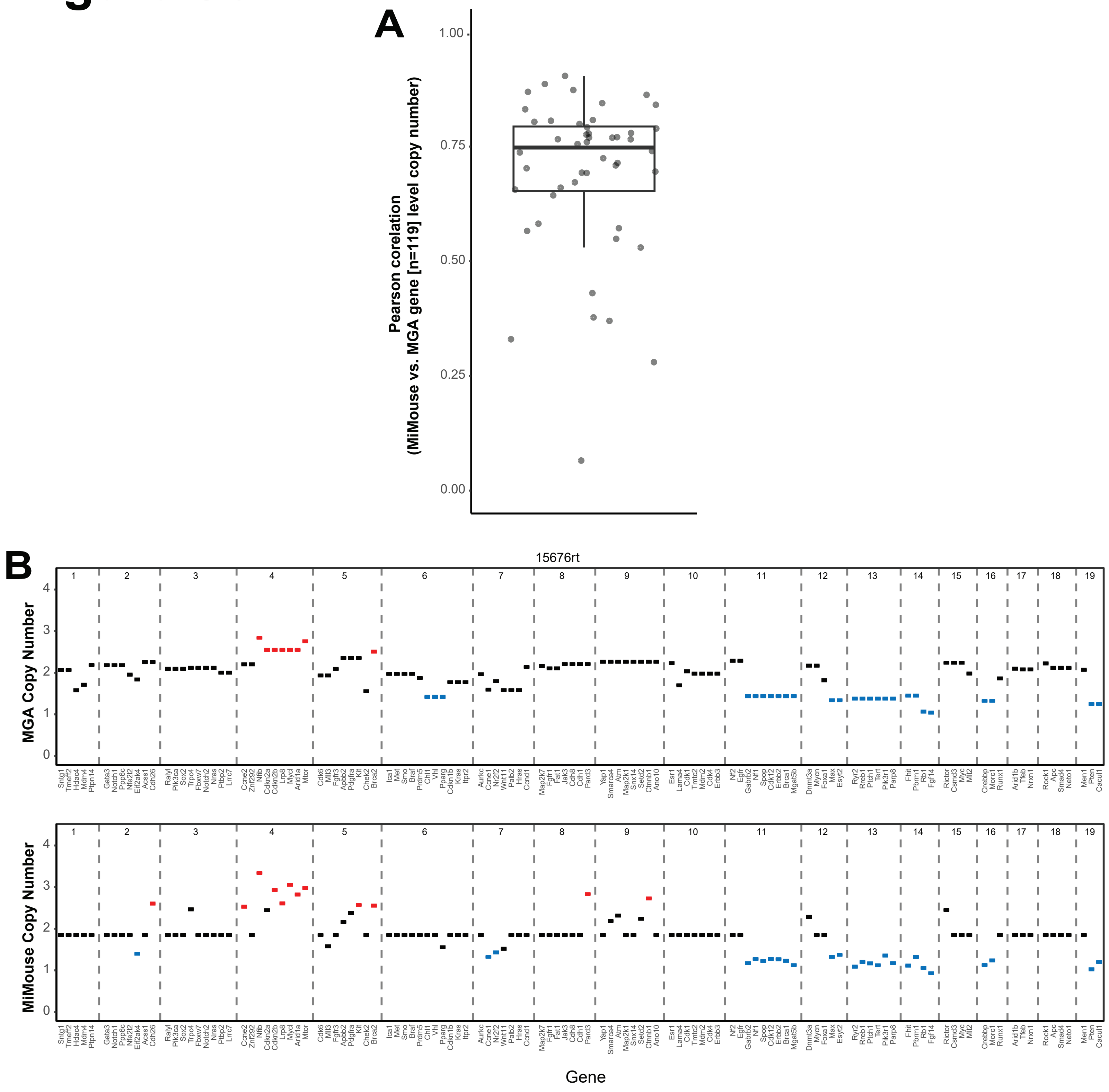

Figure S6. Gene level correlation of copy number by MiMouse and MGA.
A) Boxplot of sample level Pearson correlation of copy number for 119 genes measured on both MiMouse and MGA from samples in **Figure 3A**. **B)** Copy number plot displaying the gene-level copy numbers for the 119 genes used for comparison between MiMouse and MGA for a representative HGSC sample from **A**. Each gene (line) is plotted in genomic order and colored based on gain (red), loss (blue), or no change (black).
