# Supplementary material for: Development and Application of MiMouse, a Comprehensive Genomic Profiling Panel for Credentialing Mouse Tumor Models
Source: Cancer Res Commun. 2025 Oct 29;5(10):1910–33. doi: 10.1158/2767-9764.CRC-25-0279 (PMC12569591; doi:10.1158/2767-9764.CRC-25-0279)
Supplement: Figure S7 — Indels limit informative SNPs from Ion Torrent based MiMouse profiling [file crc-25-0279_figure_s7_suppsf7.pdf]

Figure S7

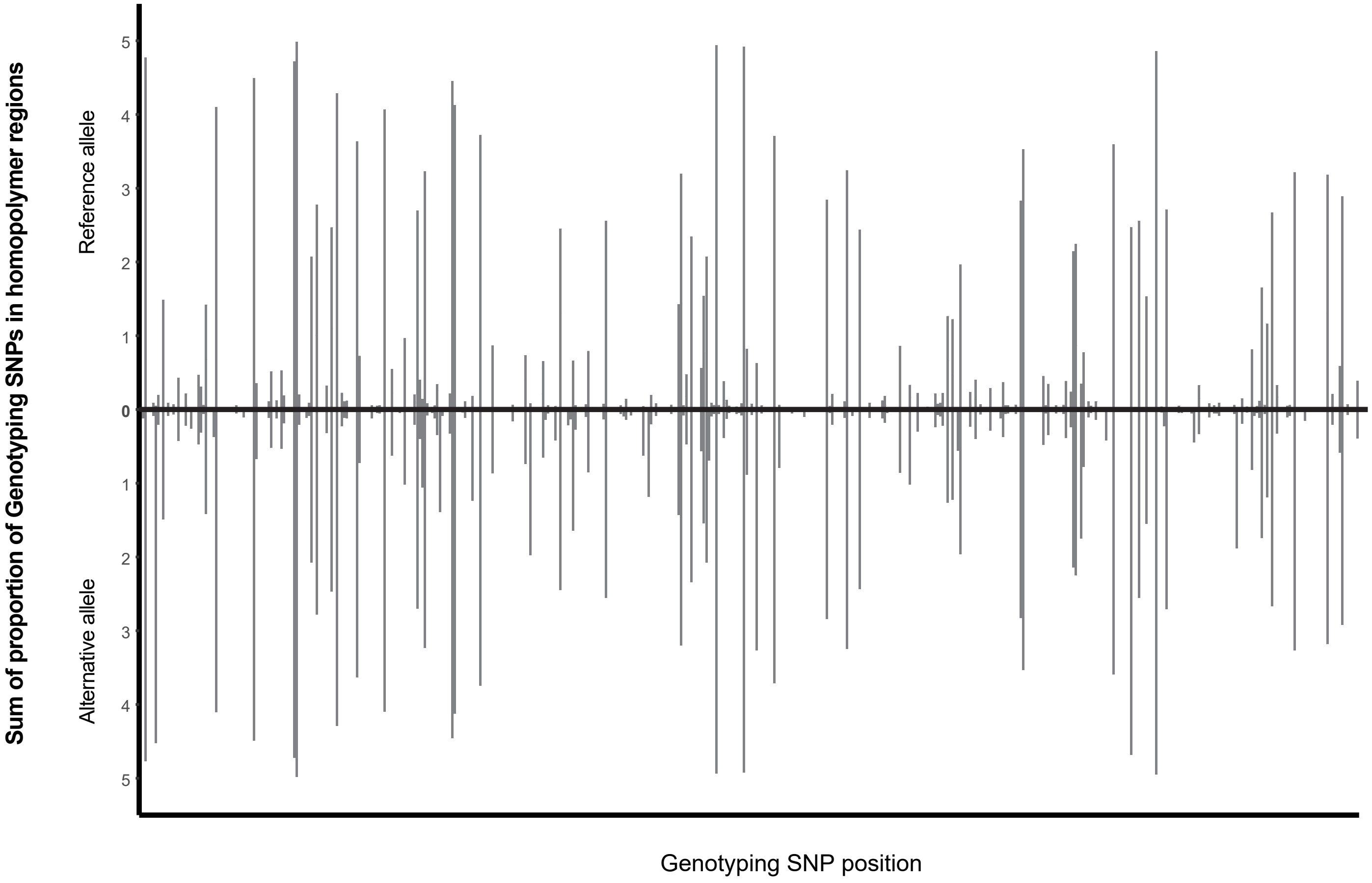

Figure S7. Indels limit informative SNPs from Ion Torrent based MiMouse profiling.
Barplot of the sum of the proportion of samples per sequencing run (n = 7) with homopolymer indels called at genomic positions with expected SNPs. Plot is separated by the indels called as a reference allele (top) and an alternative allele (bottom), and the genotyping SNP positions are sorted by genomic order.
