# Supplementary material for: Development and Application of MiMouse, a Comprehensive Genomic Profiling Panel for Credentialing Mouse Tumor Models
Source: Cancer Res Commun. 2025 Oct 29;5(10):1910–33. doi: 10.1158/2767-9764.CRC-25-0279 (PMC12569591; doi:10.1158/2767-9764.CRC-25-0279)
Supplement: Figure S8 — Highly correlated detection of focal, sub‐gene deep (homozygous) deletions by MiMouse [file crc-25-0279_figure_s8_suppsf8.pdf]

Figure S8

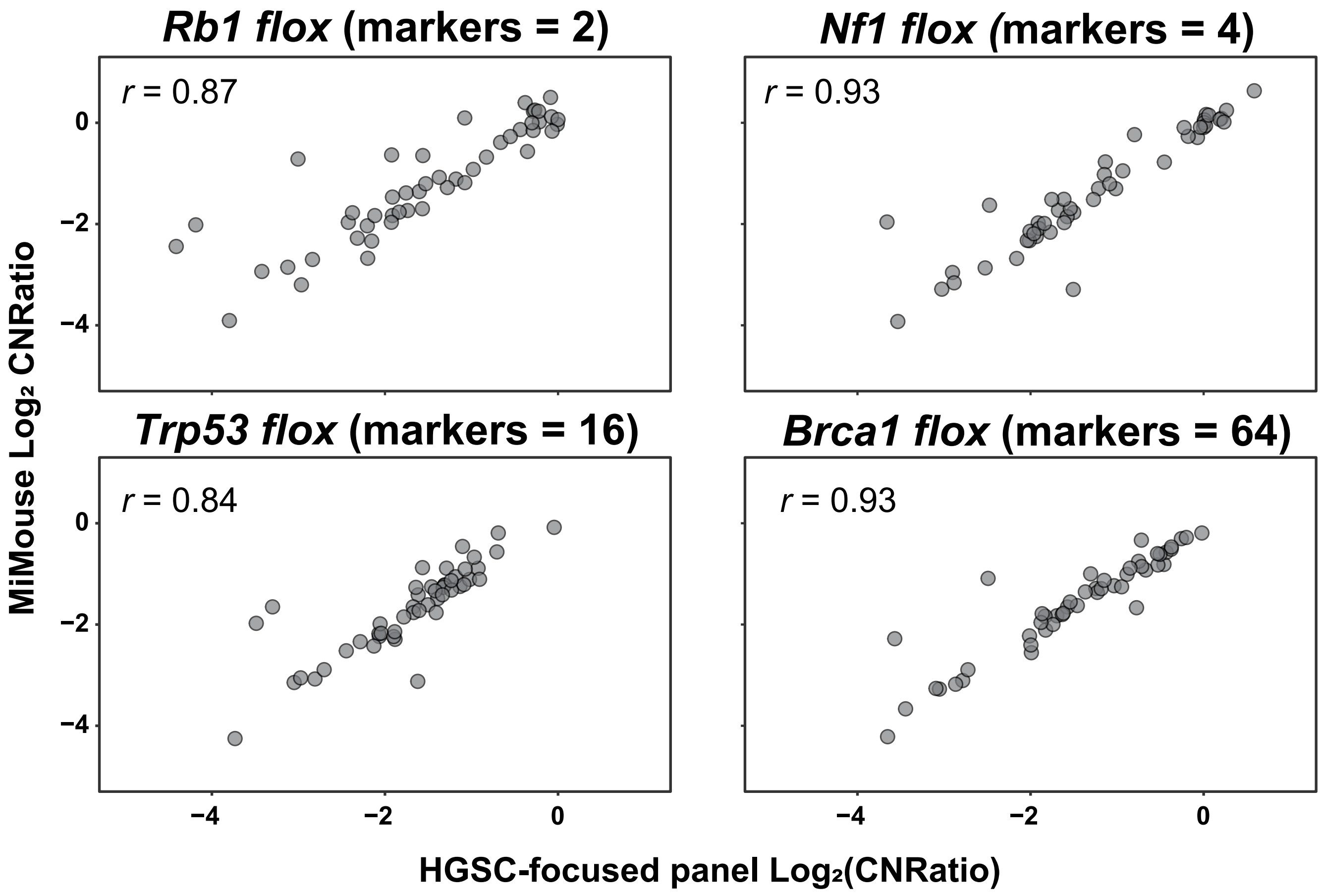

Figure S8. Highly correlated detection of focal, sub-gene deep (homozygous) deletions by MiMouse. Dot plot of log_2CNR signal of targeted flox regions for *Trp53, Brca1, Nf1 and Rb1* for 40 HGSC GEMMs (tumor content >30%) sequenced with both our previous HGSC-focused panel and MiMouse. The Pearson correlation is shown.
