# Supplementary material for: Development and Application of MiMouse, a Comprehensive Genomic Profiling Panel for Credentialing Mouse Tumor Models
Source: Cancer Res Commun. 2025 Oct 29;5(10):1910–33. doi: 10.1158/2767-9764.CRC-25-0279 (PMC12569591; doi:10.1158/2767-9764.CRC-25-0279)
Supplement: Figure S9 — MiMouse for CGP and genomic credentialling across the neoplastic spectrum of two large cohorts of inducible fallopian tube (Ovary) and colorectal (CRC) tumors [file crc-25-0279_figure_s9_suppsf9.pdf]

Figure S9

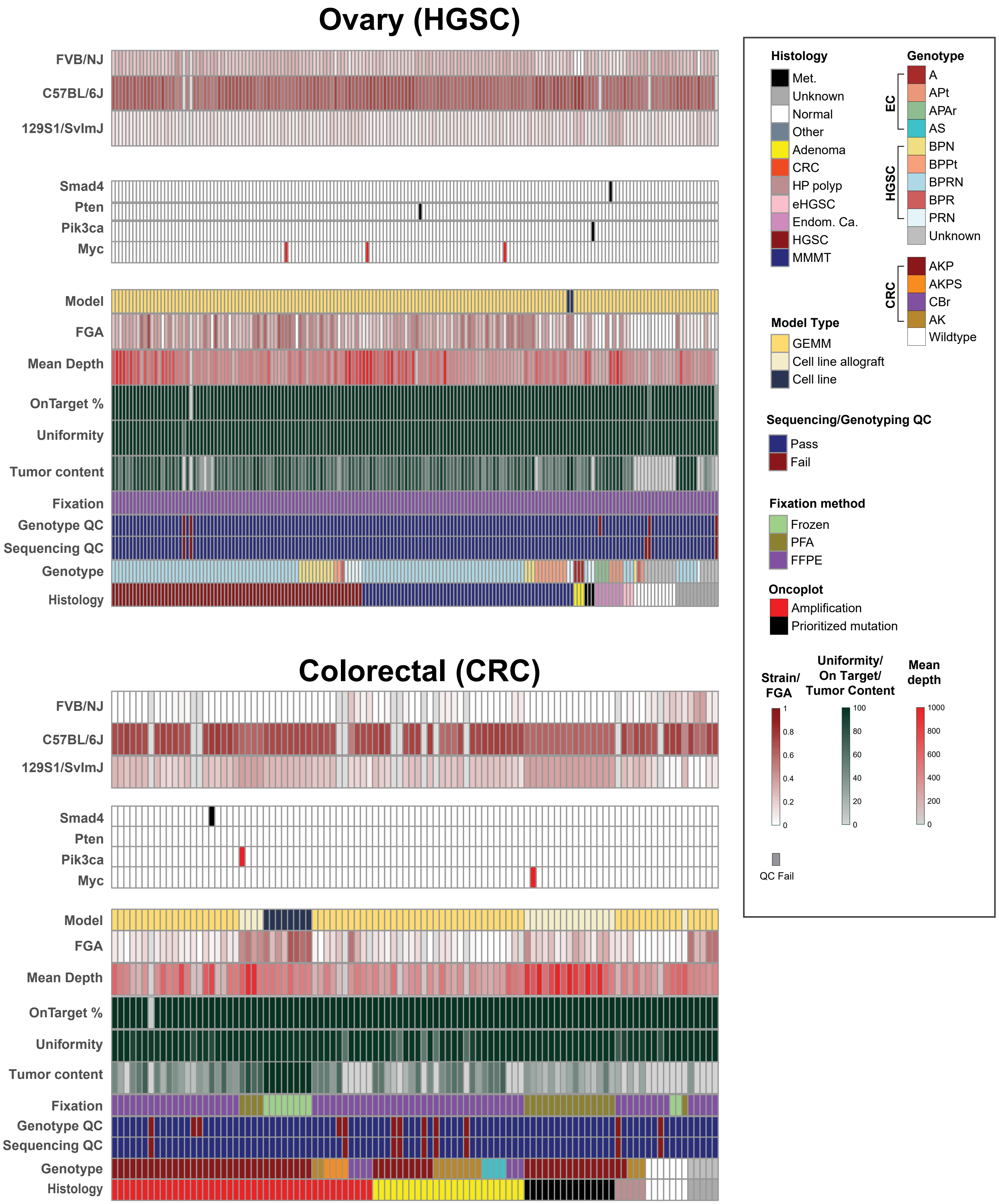
Ovary (HGSC)
FVB/NJ
C57BL/6J
129S1/SvImJ
Smad4
Pten
Pik3ca
Myc
Model
FGA
Mean Depth
OnTarget %
Uniformity
Tumor content
Fixation
Genotype QC
Sequencing QC
Genotype
Histology
Colorectal (CRC)
FVB/NJ
C57BL/6J
129S1/SvImJ
Smad4
Pten
Pik3ca
Myc
Model
FGA
Mean Depth
OnTarget %
Uniformity
Tumor content
Fixation
Genotype QC
Sequencing QC
Genotype
Histology
Histology
Met.
Unknown
Normal
Other
Adenoma
CRC
HP polyp
eHGSC
Endom. Ca.
HGSC
MMMT
Genotype
EC
A
APt
APAr
AS
HGSC
BPN
BPPt
BPRN
BPR
PRN
Unknown
CRC
AKP
AKPS
CBr
AK
Wildtype
Model Type
GEMM
Cell line allograft
Cell line
Sequencing/Genotyping QC
Pass
Fail
Fixation method
Frozen
PFA
FFPE
Oncoplot
Amplification
Prioritized mutation
Strain/ FGA
Uniformity/ On Target/ Tumor Content
Mean depth
1
0.8
0.6
0.4
0.2
0
100
80
60
40
20
0
1000
800
600
400
200
0
QC Fail

Figure S9. MiMouse for CGP and genomic credentialling across the neoplastic spectrum of two large cohorts of inducible fallopian tube (Ovary) and colorectal (CRC) tumors.

MiMouse cohort characteristics and CGP results from the Ovary (predominantly HGSC; top, total n=172) and CRC (bottom, total n=100) cohorts are shown (see **Figure 4** for details of transgenes/genotypes). Sequencing quality control (QC) is based on a combination of the sequencing uniformity, mean depth, and on target percentage. Genotyping QC is based on a sample's genotyping rate (QC pass if >80% of all genotyping SNPs called). Strain fractions were estimated using ADMIXTURE; only the three detected strains are shown (C57BL/6J, FVB/NJ and 129S1/SvImJ). Genotype (EC = endometrioid carcinoma), histology (Met. = metastasis, CRC = carcinoma, HP = hyperplastic, eHGSC = early HGSC, MMMT = carcinosarcoma) and model type (GEMM = GEMM tumor tissue) are shown. Gene-level fraction of genome altered (FGA; see main text) is shown according to the color scale. All detected somatic, prioritized, amplifications (red), deep (homozygous) deletions (blue; none detected) and prioritized somatic mutations (black) across the cohorts are shown. PFA = paraformaldehyde
