# Supplementary material for: Development and Application of MiMouse, a Comprehensive Genomic Profiling Panel for Credentialing Mouse Tumor Models
Source: Cancer Res Commun. 2025 Oct 29;5(10):1910–33. doi: 10.1158/2767-9764.CRC-25-0279 (PMC12569591; doi:10.1158/2767-9764.CRC-25-0279)
Supplement: Figure S10 — Aneuploidy events in human HGSC and CRC [file crc-25-0279_figure_s10_suppsf10.pdf]

Figure S10

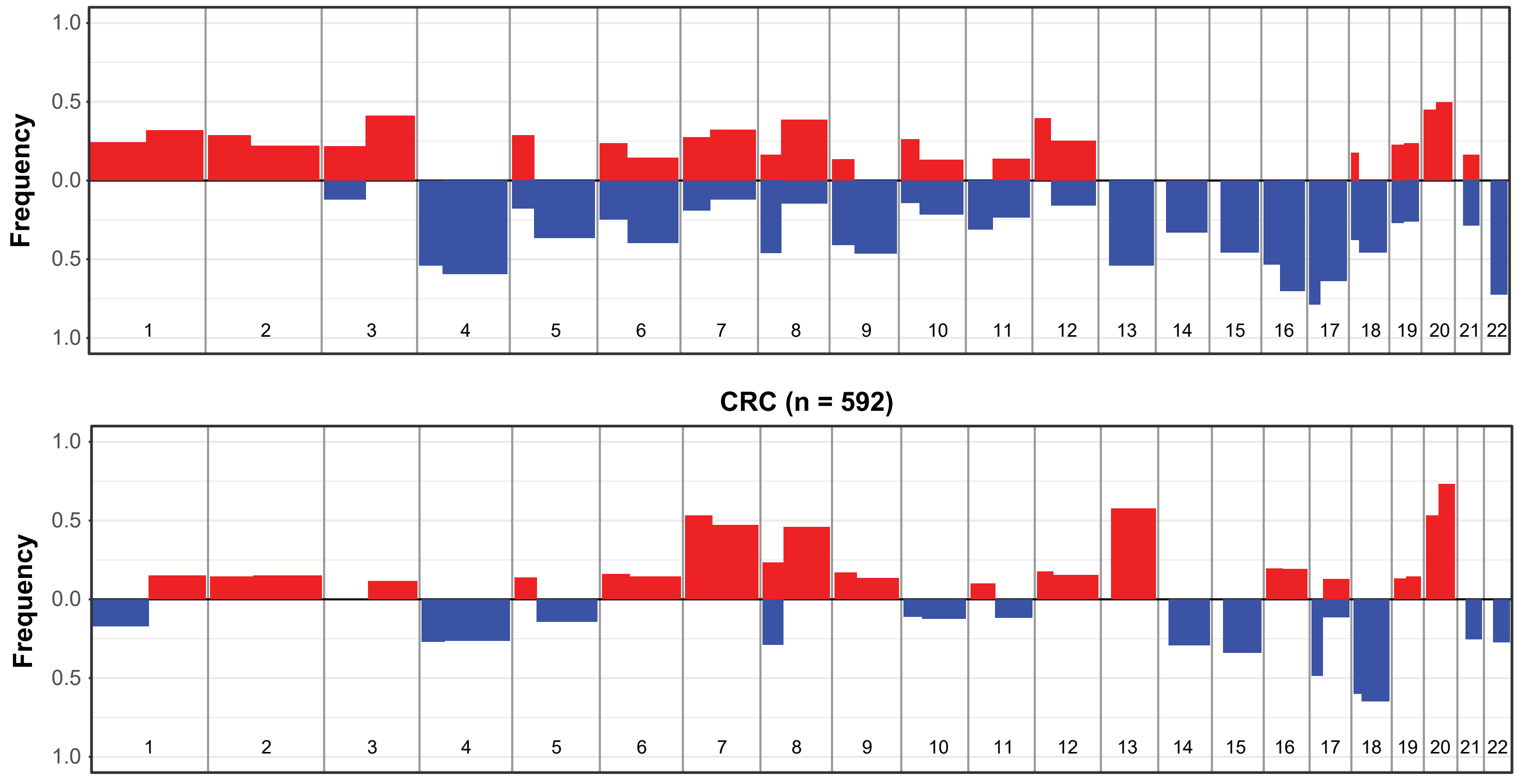

Figure S10. Aneuploidy events in human HGSC and CRC.

Frequency plots of aneuploidy in human chr arms from TCGA (using GISTIC2.0) for HGSC (n=572; top) and CRC (n = 592; bottom). Gains are in red; losses in blue. Only recurrent arm-level changes (>10%) were included.
