# Supplementary material for: Development and Application of MiMouse, a Comprehensive Genomic Profiling Panel for Credentialing Mouse Tumor Models
Source: Cancer Res Commun. 2025 Oct 29;5(10):1910–33. doi: 10.1158/2767-9764.CRC-25-0279 (PMC12569591; doi:10.1158/2767-9764.CRC-25-0279)
Supplement: Figure S11 — FGA differs in human and mouse models of HGSC and CRC [file crc-25-0279_figure_s11_suppsf11.pdf]

Figure S11

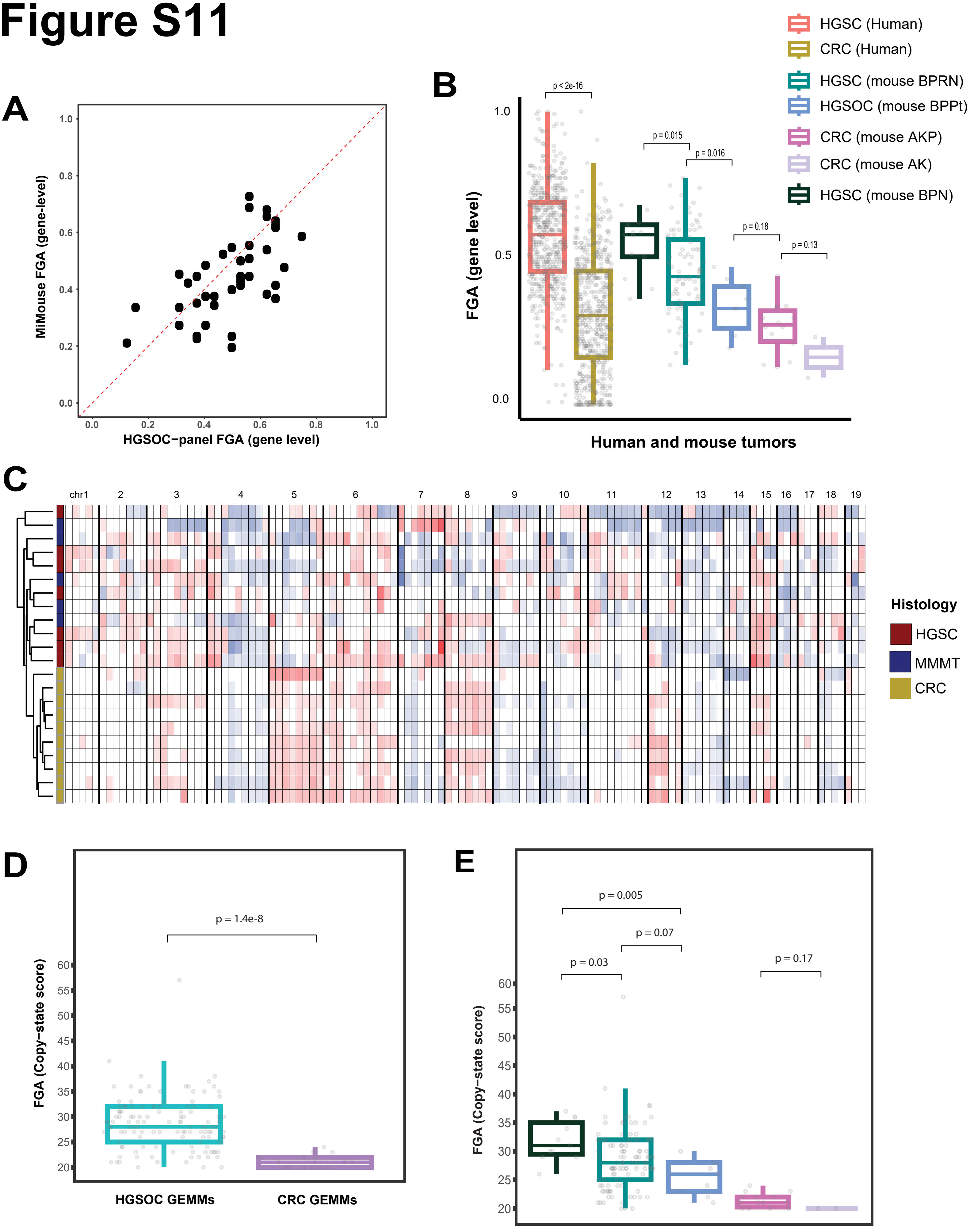

A
MiMouse FGA (gene-level)
HGSOC−panel FGA (gene level)
0.0
0.2
0.4
0.6
0.8
1.0
B
HGSC (Human)
CRC (Human)
HGSC (mouse BPRN)
HGSOC (mouse BPPt)
CRC (mouse AKP)
CRC (mouse AK)
HGSC (mouse BPN)
p < 2e-16
p = 0.015
p = 0.016
p = 0.18
p = 0.13
FGA (gene level)
1.0
0.5
0.0
Human and mouse tumors
C
chr1
2
3
4
5
6
7
8
9
10
11
12
13
14
15
16
17
18
19
Histology
HGSC
MMMT
CRC
D
p = 1.4e-8
FGA (Copy−state score)
60
55
50
45
40
35
30
25
20
HGSOC GEMMs
CRC GEMMs
E
p = 0.005
p = 0.07
p = 0.03
p = 0.17
FGA (Copy−state score)
60
55
50
45
40
35
30
25
20

Figure S11. FGA differs in human and mouse models of HGSC and CRC.

A) Comparison of gene-level fraction of the genome altered (FGA) between the same samples sequenced (n = 40) using both our previous HGSC-focused panel(7) and MiMouse (32 and 119 genes, respectively). The origin is indicated by a red dashed line. **B)** Boxplot comparing genomic instability by gene-level FGA (n=119 genes) between human (TCGA) and mouse HGSCs and CRCs (by MiMouse) with human cancers (CRC and HGSC) and mouse GEMMs, stratified by mouse genotype (see **Fig 4**). **C)** Heatmap of representative subsets of mouse CRC and HGSC samples displaying gene-level calls (log_2CNR) highlighting the qualitative difference in CNA patterns. **D)** Comparison of genomic instability in HGSC and CRC GEMMs by copy-state score FGA (sum of each chr arm's different copy-levels). Differences in the distribution were compared by Wilcoxon ranked-sum test. **(E)** As in **D**, except stratified by genotype.
