# Supplementary material for: Development and Application of MiMouse, a Comprehensive Genomic Profiling Panel for Credentialing Mouse Tumor Models
Source: Cancer Res Commun. 2025 Oct 29;5(10):1910–33. doi: 10.1158/2767-9764.CRC-25-0279 (PMC12569591; doi:10.1158/2767-9764.CRC-25-0279)
Supplement: Figure S12 — Focused NGS panels overestimate FGA vs. CGP and WES in human HGSC [file crc-25-0279_figure_s12_suppsf12.pdf]

Figure S12

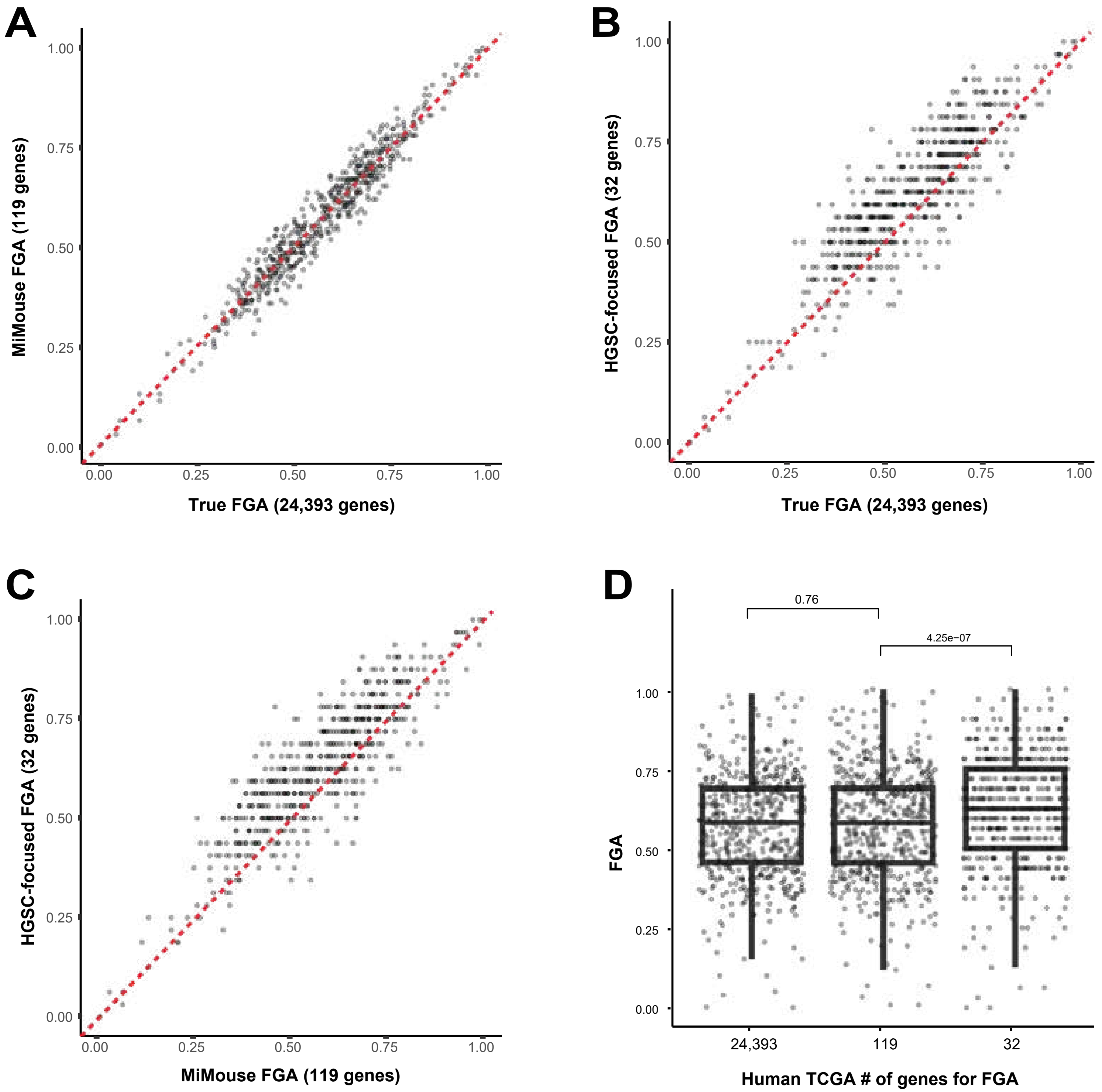

Figure S12. Focused NGS panels overestimate FGA vs. CGP and WES in human HGSC.
A&B) Comparison of FGA calculated for human HGSC samples (TCGA) using all genes (n = 24,393) vs. those on **A)** MiMouse (n=119 genes) or **B)** our HGSC-focused panel (n=32 genes). The dashed red line indicates the origin. **C)** As in **A&B**, except comparison of FGA by the HGSC-focused panel of genes vs. MiMouse. **D)** Boxplot of data from **A-C** compared by the Wilcoxon ranked-sum test.
