# Supplementary material for: Development and Application of MiMouse, a Comprehensive Genomic Profiling Panel for Credentialing Mouse Tumor Models
Source: Cancer Res Commun. 2025 Oct 29;5(10):1910–33. doi: 10.1158/2767-9764.CRC-25-0279 (PMC12569591; doi:10.1158/2767-9764.CRC-25-0279)
Supplement: Figure S13 — Comparison of aneuploidy between our HGSC models and other models [file crc-25-0279_figure_s13_suppsf13.pdf]

Figure S13

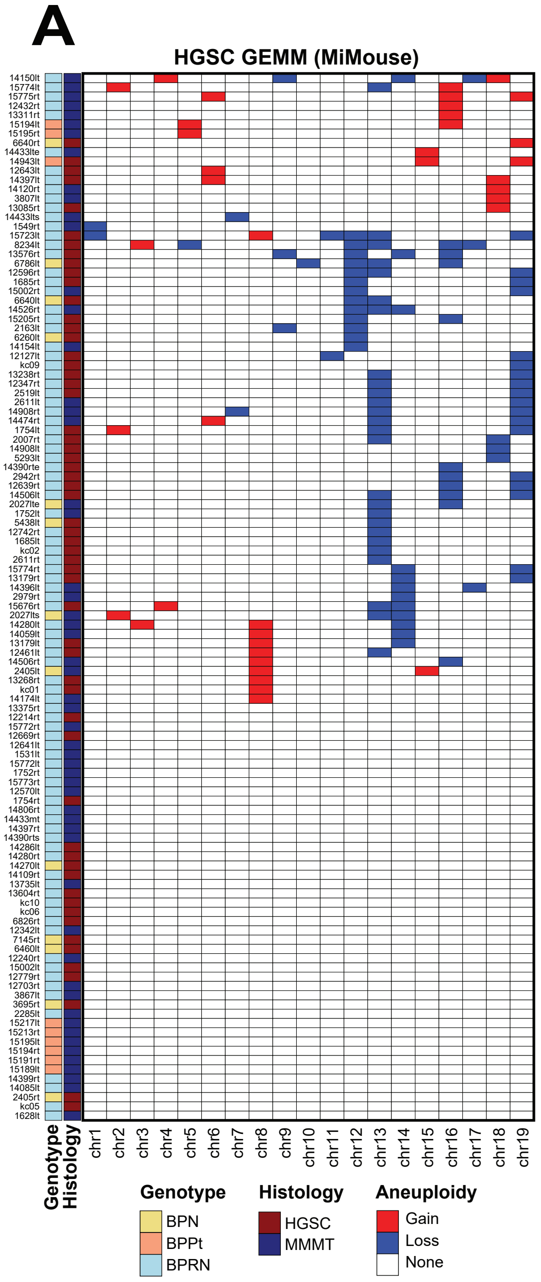
A
HGSC GEMM (MiMouse)
Genotype
Histology
chr1 chr2 chr3 chr4 chr5 chr6 chr7 chr8 chr9 chr10 chr11 chr12 chr13 chr14 chr15 chr16 chr17 chr18 chr19
Genotype
BPN
BPPt
BPRN
Histology
HGSC
MMMT
Aneuploidy
Gain
Loss
None

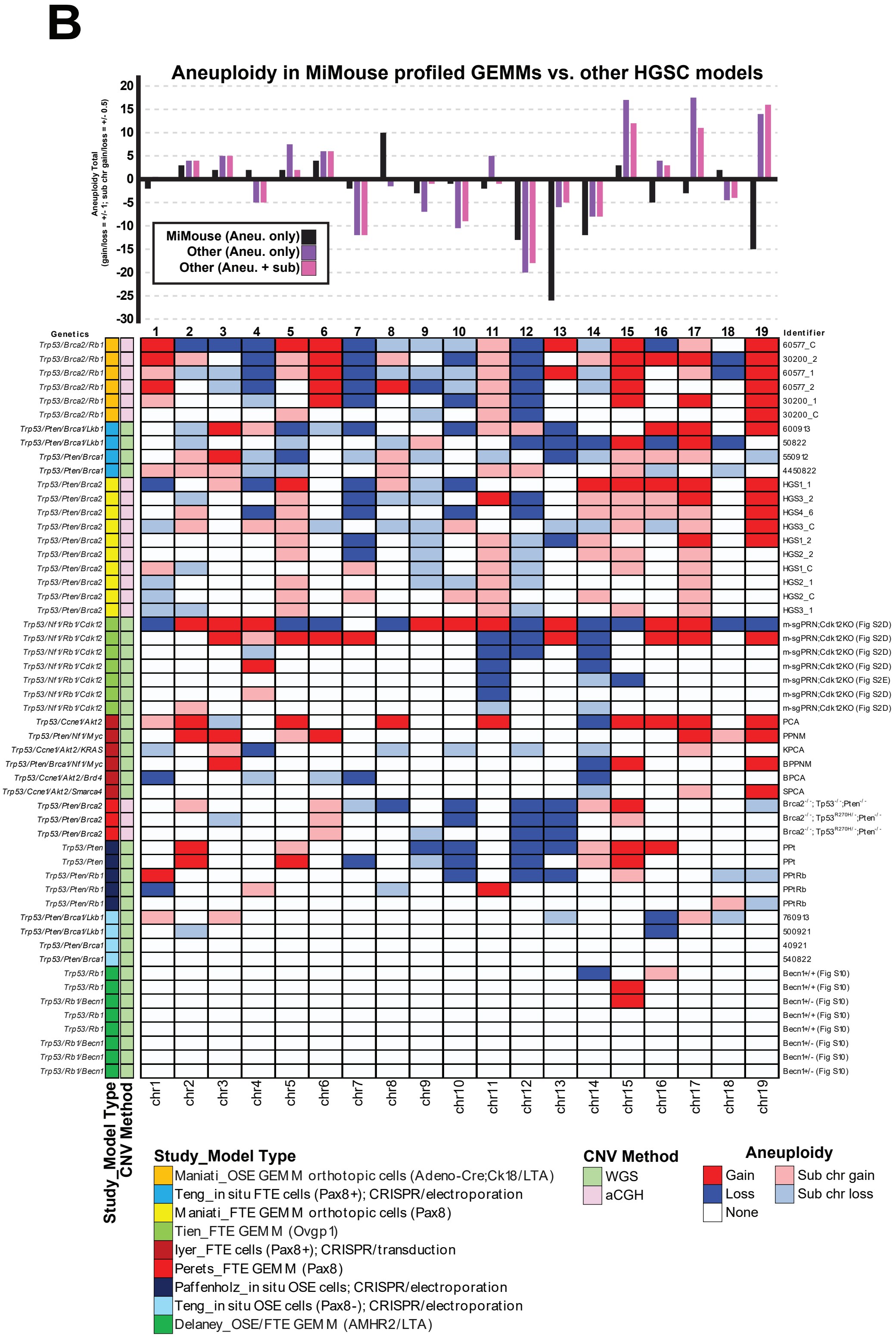
B
Aneuploidy in MiMouse profiled GEMMs vs. other HGSC models
Aneuploidy Total (gain/loss = +/- 1; sub chr gain/loss = +/- 0.5)
MiMouse (Aneu. only)
Other (Aneu. only)
Other (Aneu. + sub)
Genetics
Identifier
Study_Model Type
CNV Method
Maniati_OSE GEMM orthotopic cells (Adeno-Cre;Ck18/LTA)
Teng_in situ FTE cells (Pax8+); CRISPR/electroporation
Maniati_FTE GEMM orthotopic cells (Pax8)
Tien_FTE GEMM (Ovgp1)
Iyer_FTE cells (Pax8+); CRISPR/transduction
Perets_FTE GEMM (Pax8)
Paffenholz_in situ OSE cells; CRISPR/electroporation
Teng_in situ OSE cells (Pax8-); CRISPR/electroporation
Delaney_OSE/FTE GEMM (AMHR2/LTA)
CNV Method
WGS
aCGH
Aneuploidy
Gain
Loss
None
Sub chr gain
Sub chr loss

Figure S13. Comparison of aneuploidy between our HGSC models and other models.
A) Heatmap showing aneuploidy status of each mouse chromosome for HGSC GEMM tumor samples (n=113) profiled by MiMouse. Genotype, histology, and aneuploidy events are indicated according to the legend. **B**) Aneuploidy status was determined by visual evaluation from published genome-wide copy number plots from individual samples (n=53) in 7 studies of HGSC models(8-14) and shown in a heatmap. Sub-chromosome (chr) level gains/losses are indicated in lighter hues. The genetics (genes manipulated) and the identifier from the published study are indicated; the first author of the study/ model type (Study_Model Type) and the CNV profiling method (array CGH [aCGH] or whole genome sequencing [WGS]) are indicated according to the legend. To compare aneuploidy profiles between our MiMouse profiled HGSC models (black) to those from the other studies, in each cohort, aneuploidy events (gain or loss = +/-1) per chromosome arm were summed and the total plotted (for the other cohorts, the total was determined both excluding [purple] and including [pink] sub-chromosome level events [sub-chr gain or loss = +/- 0.5]).
