# Supplementary material for: Development and Application of MiMouse, a Comprehensive Genomic Profiling Panel for Credentialing Mouse Tumor Models
Source: Cancer Res Commun. 2025 Oct 29;5(10):1910–33. doi: 10.1158/2767-9764.CRC-25-0279 (PMC12569591; doi:10.1158/2767-9764.CRC-25-0279)
Supplement: Figure S14 — CRC tumors from AK and AKP mice show somatic loss of the second Apc allele [file crc-25-0279_figure_s14_suppsf14.pdf]

# Figure S14

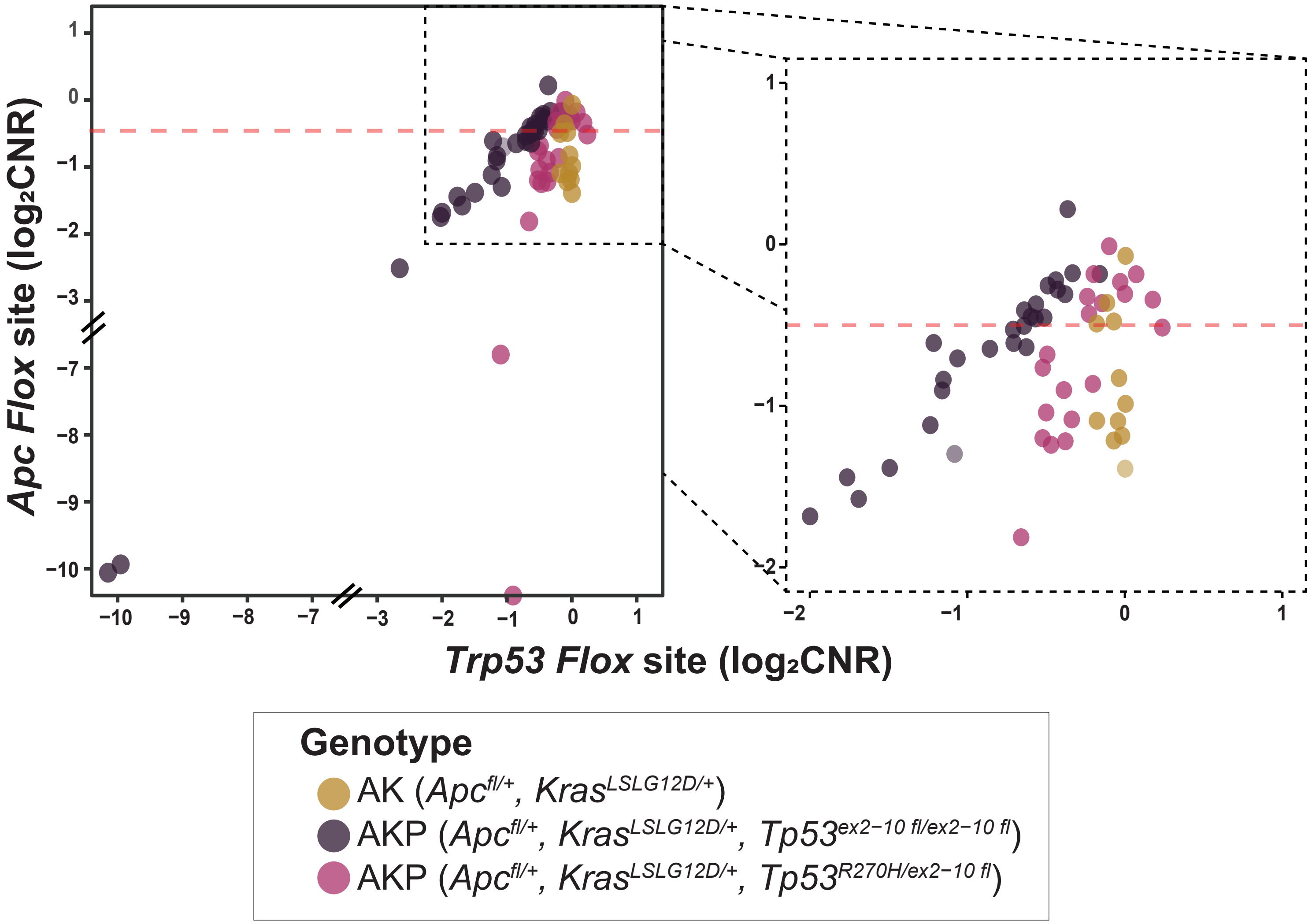


**Figure S14. CRC tumors from AK and AKP mice show somatic loss of the second *Apc* allele.**
**A)** Comparison of $log_2$CNR of the *Trp53* and *Apc* targeted flox regions for CRC samples passing sequencing QC. Samples are colored by genotype. The red dotted line denotes the required minimum tumor content (>30%) for inclusion.
