# Supplementary material for: Development and Application of MiMouse, a Comprehensive Genomic Profiling Panel for Credentialing Mouse Tumor Models
Source: Cancer Res Commun. 2025 Oct 29;5(10):1910–33. doi: 10.1158/2767-9764.CRC-25-0279 (PMC12569591; doi:10.1158/2767-9764.CRC-25-0279)
Supplement: Figure S15 — Aneuploidy comparison between HGSC and CRC models [file crc-25-0279_figure_s15_suppsf15.pdf]

Figure S15

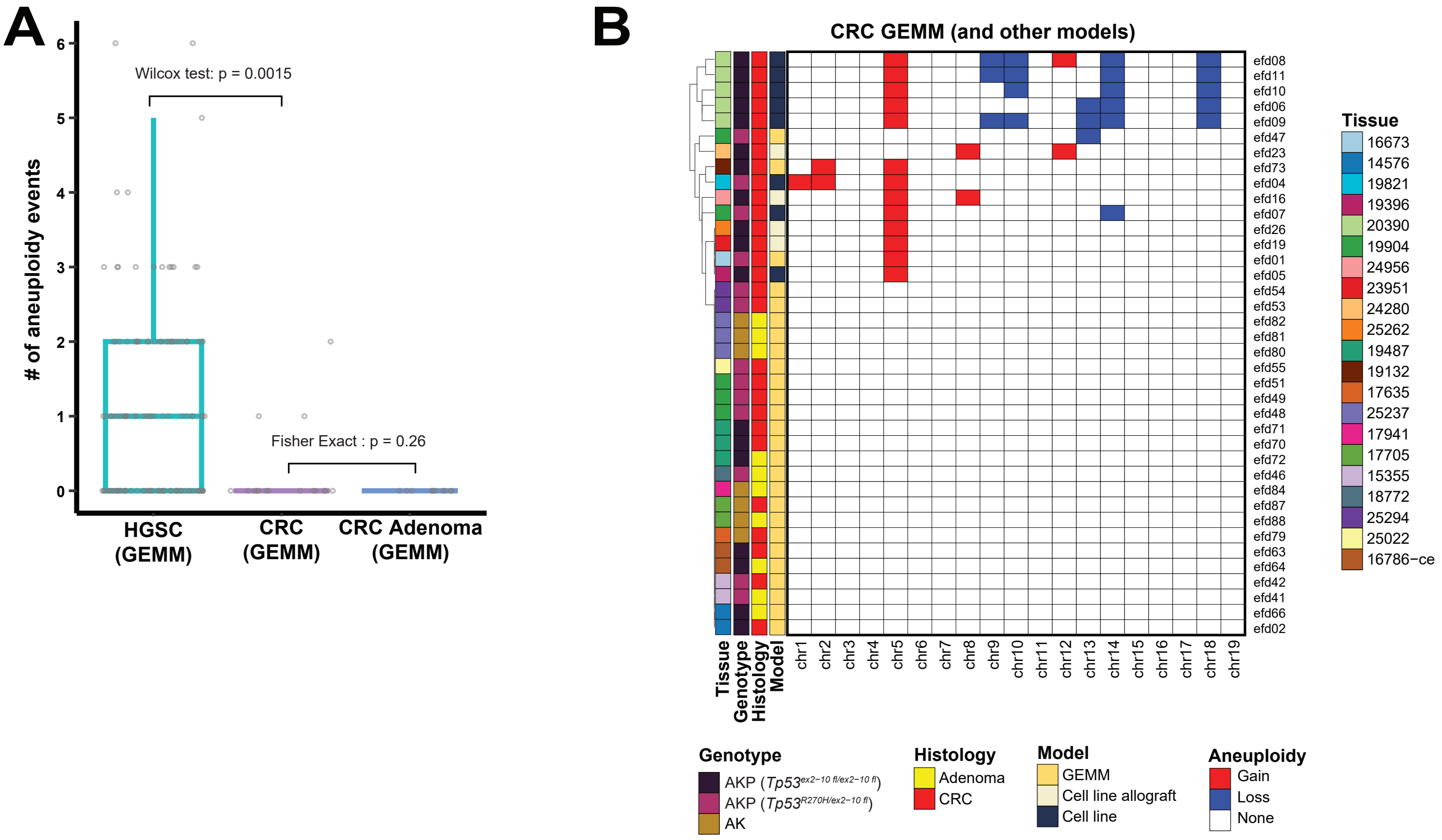

Figure S15. Aneuploidy comparison between HGSC and CRC models.
A) The number of aneuploidy events per GEMM HGSC (n = 113), CRC carcinoma (n=16) and CRC adenoma (n=10) samples are shown, with the indicated statistical comparisons. **B)** Heatmap showing aneuploidy status of each mouse chromosome arm for CRC tumor samples, with sample level hierarchical clustering.
