# Supplementary material for: Development and Application of MiMouse, a Comprehensive Genomic Profiling Panel for Credentialing Mouse Tumor Models
Source: Cancer Res Commun. 2025 Oct 29;5(10):1910–33. doi: 10.1158/2767-9764.CRC-25-0279 (PMC12569591; doi:10.1158/2767-9764.CRC-25-0279)
Supplement: Figure S16 — Contribution of aneuploidy vs. focal events to FGA in human and mouse CRC and HGSC [file crc-25-0279_figure_s16_suppsf16.pdf]

Figure S16

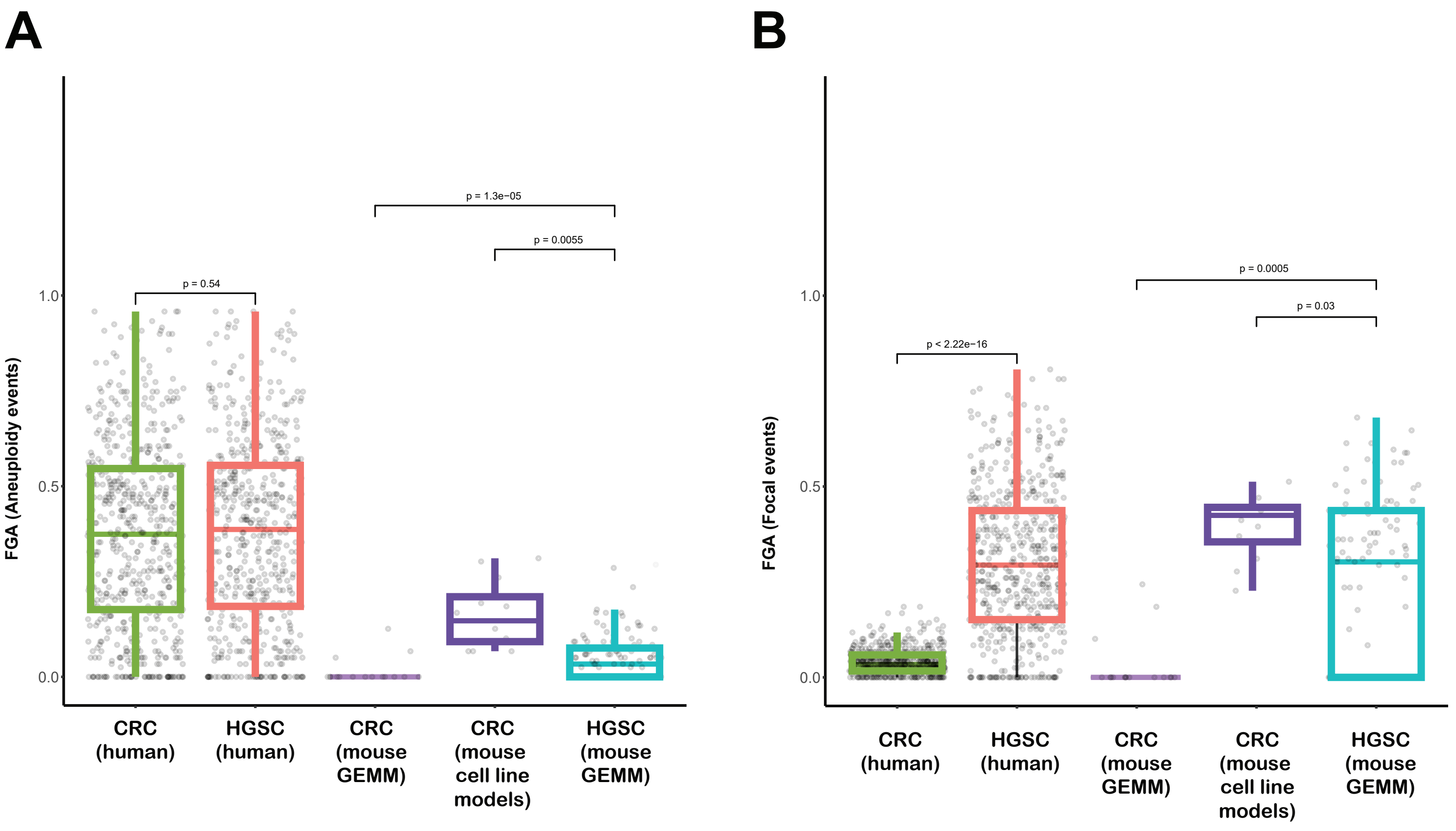

Figure S16. Contribution of aneuploidy vs. focal events to FGA in human and mouse CRC and HGSC. **A&B)** Comparison of FGA contributed by **(A)** aneuploidy events **(B)** focal event in human (TCGA) CRC and HGSC, as well as MiMouse profiled CRC (GEMM vs. cell lines) and HGSC (GEMM). Distributions within a species were compared by Wilcoxon rank sum tests.
