# Supplementary material for: Development and Application of MiMouse, a Comprehensive Genomic Profiling Panel for Credentialing Mouse Tumor Models
Source: Cancer Res Commun. 2025 Oct 29;5(10):1910–33. doi: 10.1158/2767-9764.CRC-25-0279 (PMC12569591; doi:10.1158/2767-9764.CRC-25-0279)
Supplement: Figure S17 — Comparison of dependency scores and RNA expression for syntenic region 13q12.2 [file crc-25-0279_figure_s17_suppfs17.pdf]

Figure S17

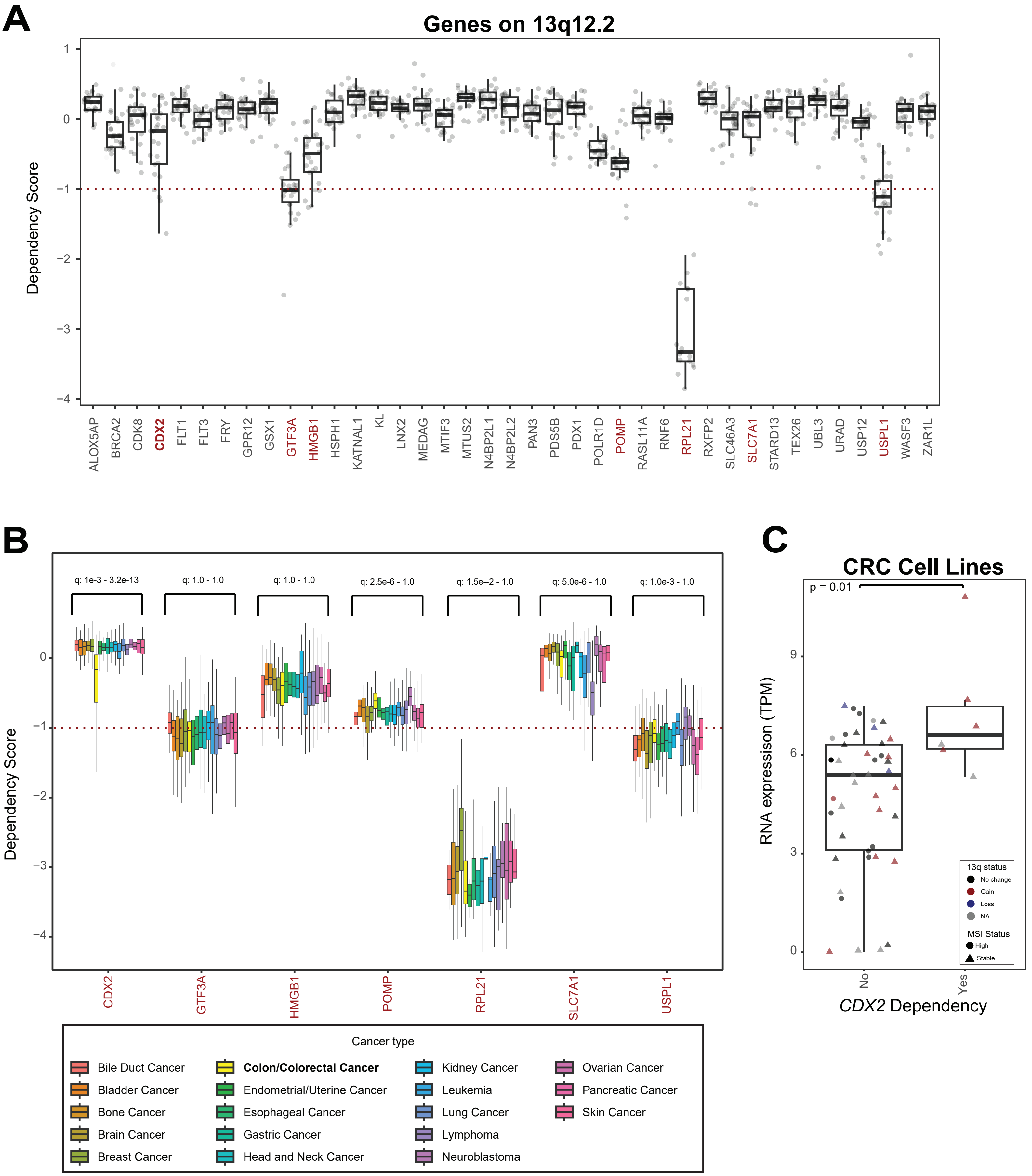

A
Genes on 13q12.2
Dependency Score
1
0
−1
−2
−3
−4
ALOX5AP
BRCA2
CDK8
CDX2
FLT1
FLT3
FRY
GPR12
GSX1
GTF3A
HMGB1
HSPH1
KATNAL1
KL
LNX2
MEDAG
MTIF3
MTUS2
N4BP2L1
N4BP2L2
PAN3
PDS5B
PDX1
POLR1D
POMP
RASL11A
RNF6
RPL21
RXFP2
SLC46A3
SLC7A1
STARD13
TEX26
UBL3
URAD
USP12
USPL1
WASF3
ZAR1L
B
q: 1e-3 - 3.2e-13
q: 1.0 - 1.0
q: 1.0 - 1.0
q: 2.5e-6 - 1.0
q: 1.5e--2 - 1.0
q: 5.0e-6 - 1.0
q: 1.0e-3 - 1.0
Dependency Score
0
−1
−2
−3
−4
CDX2
GTF3A
HMGB1
POMP
RPL21
SLC7A1
USPL1
Cancer type
Bile Duct Cancer
Bladder Cancer
Bone Cancer
Brain Cancer
Breast Cancer
Colon/Colorectal Cancer
Endometrial/Uterine Cancer
Esophageal Cancer
Gastric Cancer
Head and Neck Cancer
Kidney Cancer
Leukemia
Lung Cancer
Lymphoma
Neuroblastoma
Ovarian Cancer
Pancreatic Cancer
Skin Cancer
C
CRC Cell Lines
p = 0.01
RNA expresison (TPM)
9
6
3
0
No
Yes
CDX2 Dependency
13q status
No change
Gain
Loss
NA
MSI Status
High
Stable

Figure S17. Comparison of dependency scores and RNA expression for syntenic region 13q12.2.
A) Comparison of dependency scores from depmap CRISPR assays for all 39 genes residing on 13q12.2 (the syntenic region between human chr 13 and mouse chr 5). Red line indicates the threshold of ≤ -1 for genes to be considered essential; those genes passing this threshold in any cell line are highlighted in red. **B)** Comparison of the seven genes found to be essential in at least one cell line, stratified by CRC (colon/colorectal cancer; yellow) vs. 17 other tumor types. The range of q-values from pairwise Wilcoxon ranked sum test between the CRC cell lines and the cell lines of other cancer types are listed above each gene. **C)** RNA expression (in transcripts per million) from depmap for *CDX2* in CRC cell lines stratified by dependency score ≤ -1 (Yes; 6/7 with expression data) or > -1 (No). Cell lines with and without dependency scores ≤ -1 were compared by a Wilcoxon ranked sum test. MSI statuses of high (circle) and stable (triangle) is based on the MSIsensor2 score threshold of >= 20; the aneuploidy status of 13q, when available, is stratified by color.
